# School burnout trends and sociodemographic factors in Finland 2006–2019

**DOI:** 10.1007/s00127-022-02268-0

**Published:** 2022-03-22

**Authors:** Sanna Read, Lauri Hietajärvi, Katariina Salmela-Aro

**Affiliations:** 1 Care Policy and Evaluation Centre, London School of Economics and Political Science, Houghton Street, London, WC2A 2AE UK; 2 University of Helsinki, Helsinki, Finland

**Keywords:** Burnout, Secondary school, Sociodemographic, Trend

## Abstract

**Purpose:**

To identify the changes of school burnout for Finnish adolescents in lower (grades 8–9) and upper secondary schools (grades 10–11) during years 2006–2019; and to examine the associations of personal—(gender, family socioeconomic, and immigrant status) and school-related (school level, urban–rural area) sociodemographic demands and resources in school burnout.

**Methods:**

We used nationally representative data on 949,347 students in secondary school in Finland between 2006 and 2019. Generalized Linear Models were used to assess the effects of year, gender, school level, parental education, unemployment, immigrant status, and urban–rural area and the interactions of year, gender, and school level with each of the remaining sociodemographic variables on school burnout.

**Results:**

School burnout increased among girls and slightly declined among boys. The increase intensified in girls and the decline in boys stagnated after 2011. The educational level of the parents had a constant protective impact over time, the gradient for boys slightly larger compared to girls. Urban areas contributed to the trend of increasing school burnout among girls but not among boys. Parental unemployment and immigration background were associated with the increasing trend of school burnout over time, although somewhat mitigated by parental education.

**Conclusion:**

The results showed the trends in school burnout are often gendered and appeared to worsen aligned with the school budget cuts after 2011. In addition to considering school burnout related to lower parental education and urbanization, it is important to support those students in families experiencing unemployment and/or immigration, especially when concurring with lower parental education.

**Supplementary Information:**

The online version contains supplementary material available at 10.1007/s00127-022-02268-0.

## Introduction

Many policymakers and educators focus on enhancing youth’s emotional engagement in school to address issues of underachievement, truancy, and school dropout [1]. However, no study has examined the trends in school burnout over a longer time and how the concurrent trends in socioeconomic factors are associated with school burnout among lower and upper secondary school students. School burnout, defined as students’ exhaustion, cynicism about the value of school, and feeling of inadequacy to be successful, influences students’ engagement with schoolwork, well-being, and adjustment [2]. Currently, our understanding is limited in part by the fact that most of the research in the emotional engagement at school focuses on adolescents in the United States [3], where many students experience declines in emotional engagement, as well as academic and psychological outcomes, over the course of secondary school [4]. Studying changes in school burnout in a country such as Finland—where students attain consistently high levels of academic achievement throughout secondary school despite recent evidence showing that students may not enjoy school—could provide some unique insights into the issue of student burnout. Moreover, changing socioeconomic trends, such as larger proportions of adults gaining higher education degrees, increasing immigration, urbanization and unemployment, and the concurrent policy changes, such as the education budget cuts in the past years, may also affect the patterns of school burnout.

In 1970, the government of Finland decided to overhaul its traditional education system in favour of a “modern, publicly financed education system with widespread equity, good quality, and large participation—all at a reasonable cost” [5]. After the reform, Finnish students became one of the best performers on the PISA (Programme for International Student Assessment), consistently achieving top scores in mathematics, science, and reading. However, the evidence also shows that Finnish adolescents may not be emotionally engaged in school. The 2012 PISA results reveal that 15-year-old Finnish students ranked 61st out of 65 countries for how happy they feel at school [6]. Many Finnish secondary school students report school burnout [7]. While evidence of school burnout among Finnish youth is mounting, researchers have yet to investigate the trends in school burnout both in the lower and upper secondary schools during the last decades and to what extent personal and school-related demands and resources explain these changes [8]. The current study can shed light on whether school burnout differs by time, educational contexts, or gender, and whether the socioeconomic demands and resources play a role in trends in school burnout. Most importantly, this study can help us determine the extent to which the trend of school burnout is modified by the socioeconomic demands and resources, informing the design for targeted interventions.

According to the demands-resources model in the school context [8], personal- and school-related demands, and resources influence school burnout. The more both school and personal demands, the more school burnout the students experience and, in turn, the more resources, the less school burnout the students experience. In addition, higher resources can attenuate the effects of higher demands, that is, the more resources a student can capitalize on, the more demands can be handled without overtaxing. For instance, the protective effect of higher educational level of the parents may buffer against the possible adverse effect of parental unemployment (or immigration). In the current study, we focus on the sociodemographic demands and resources: person-related (gender, immigration status, parental education, and unemployment) and school-related (school level and urban–rural area).

Of the person-related factors, the previous research shows that school burnout is higher among girls compared to boys in the secondary school [9]. When following up the same students in secondary school in Finland, school burnout has been found to be increasing [10–12], especially among girls [11]. Both female gender and low academic performance have been associated with school burnout [13]. Although girls often show better school performance compared to boys suggesting a lower likelihood of burnout, girls may experience more pressures related to ambitious educational and occupational goals in association with higher academic attainment and aspirations [14]. These, in turn, may contribute to school burnout.

Within the family, parents burnout and their children’s burnout have been shown to be shared [15]. The more economic hardship there is in the family, the more burnout in the family which may contribute to school burnout in children [16, 17]. Parental unemployment, immigration, and urban area may contribute to higher stress levels and burnout [11, 18]. A higher parental educational level in turn may act as a buffer against school burnout [11], and may also alleviate the adverse effects of the other demands.

These socioeconomic factors were also known to change over the time of the study 2006–2019. The great recession hit globally during 2008 and beyond, but in Finland, the economic crisis hit later: the peak of the unemployment (10.2% in men and 9.0% in women) was in 2015, while in 2019, the rates were about the same level as in 2006 (7.4% in men and 6.3% in women in 2019 vs. 7.5% in men and 8.1% in women in 2006) [19]. In addition to unemployment, Finland has also experienced small but increasing numbers of individuals and families immigrating to Finland with a peak of 34,900 immigrants in 2016 [20]. About 8% of the population in Finland in 2019 had a foreign background. A recent entry to the country, especially among boys, has been found to be associated with school burnout [16]. Finland like many other Western countries has also experienced internal migration from rural to urban areas. In 2006, 34% of the 15–18 years old lived in rural areas in Finland, but in 2019, this proportion had dropped to 28% [21]. At the same time, educational level has increased in Finland: a larger proportion had attained a higher education degree in 2019 compared to 2007 (47 vs. 39% among those aged 40–49) [22]. To take into account these trends, we included the effect of the year of the survey on parental unemployment, immigration status, urban–rural area, and parental educational level in the models. We also investigate whether the school burnout trends coincide with school budget cuts carried out from 2011 onwards [23], totaling about €1.5 billion, and potentially jeopardising equal access, quality, and quantity of teaching and affecting students’ well-being.

The aim was to examine changes in school burnout using data among almost one million students during the last two decades:

- (a) To identify the changes of school burnout for Finnish adolescents in lower (grades 8–9) and upper secondary schools (grades 10–11) during years 2006–2019. Based on the findings from the previous research, we expect that the overall trend of school burnout over the period from 2006 to 2019 would be increasing, especially from 2011 onwards along with the school budget cuts.
- (b) To examine the associations of personal—(gender, family socioeconomic, and immigrant status) and school-related (school level and urban–rural area) sociodemographic demands and resources with school burnout. School burnout has been found to be gendered and vary between the school levels [9–12]. We expect high and increasing school burnout, especially in girls in upper secondary school. Personal demands related to parental unemployment, immigration status, and urban–rural area are expected to be associated with higher school burnout. Parents’ higher educational degrees may in turn act as a buffer against school burnout. Although there is no previous research specifically on the interactions of gender, school level, and other socioeconomic factors on school burnout trend, it is possible that those groups who have experiences increasing school burnout (girls, upper secondary school) would be more affected by adverse socioeconomic circumstances but also may benefit from family resources such as higher parental education.

## Method

### Sample and data collection

We used the data from the Finnish School Health Promotion Survey (SHPS) [24]. The SHPS is a bi-annual nationwide classroom survey to monitor the health and well-being of Finnish 14–18-year-old adolescents. The survey was confidential and anonymous, and participation was voluntary. Participants gave informed consent by answering the survey. The parents of the participants who were under 15 years were informed about the survey in advance and had an option to withdraw the consent to participate. Ethical approval for SHPS was given by the Finnish National Institute for Health and Welfare.

The current study used data collected between 2006 and 2019. The data comprised a representative sample of 631,166 students in lower secondary schools and 318,181 students in upper secondary schools, a total of 949,347 students (70–80% of the yearly student population). Special educational schools and schools with less than 10 students were not included. The proportions of the participants by the study year are shown in Online Resource 1. There was a gradual decline in the response rate from 2006 to 2019. The students in lower secondary school were 14–16 years, and in upper secondary school 16–18 years. The distribution of the SES, immigrant status, and urban–rural are shown in Tables 1 and 2.

**Table 1:** Distributions of the variables in the School Health Promotion Study 2006–2019 in girls and boys in lower and upper secondary school

Variable	Girls—lower		Girls—upper		Boys—lower		Boys—upper	
	%/Mean (SD)	% Missing	% or Mean (SD)	% Missing	% or Mean (SD)	% Missing	% or Mean (SD)	% Missing
School burnout	*n* = 314,468		*n* = 184,001		*n* = 313,601		*n* = 133,464	
	1.8 (0.75)	0.2	2.0 (0.70)	0.1	1.9 (0.74)	0.5	1.7 (0.65)	0.2
Urban–rural area	*n* = 315,174		*n* = 184,148		*n* = 315,214		*n* = 133,717	
Urban	66.1	0	73.3	0	65.7	0	73.3	0
Semi-urban	17.8	0	14.5	0	18.0	0	14.9	0
Rural	16.1	0	12.2	0	16.2	0	11.8	0
Parent(s) with HE degree	*n* = 289,928		*n* = 181,670		*n* = 289,928		*n* = 130,935	
	41.4	5.7	53.3	1.3	43.5	8.0	60.1	2.1
Parent(s) unemployed	*n* = 271,113	1.4	*n* = 157,907	0.6	*n* = 272,249	3.0	*n* = 115,460	1.0
None	70.8		74.2		72.6		76.1	
One	26.0		23.0		24.0		21.5	
Two or more	3.3		2.5		3.3		2.4	
Immigration status	*n* = 155,456	2.1	*n* = 96,858	0.7	*n* = 154,611	5.8	*n* = 69,395	1.7
Native	87.9		90.0		87.4		89.7	
One parent foreign-born	7.3		6.3		6.5		6.2	
Foreign-born parents, born in Finland	2.0		1.7		1.9		1.7	
Foreign-born parents, born abroad	2.8		2.0		4.2		2.3

**Table 2:** Distributions (%) of parental education, urban area, parental unemployment, and family immigration status in the School Health Promotion Study 2006–2019

Year of survey	Parent(s) with higher degree	Urban area	One unemployed parent	Two + unemployed parents	One parent foreign-born	Foreign-born parents, born in Finland	Foreign-born parents, born abroad
2006–07	38.4	66.2	20.2	2.3	–	–	–
2008–09	42.4	67.7	22.3	2.7	–	–	–
2010–11	42.3	68.6	26.2	3.4	–	–	–
2013	50.5	69.4	24.5	3.0	6.0	1.6	2.5
2015	51.2	70.0	27.2	3.6	6.5	1.9	3.1
2017	53.2	67.5	26.8	3.6	7.0	1.9	3.3
2019	58.4	70.1	–	–	7.4	2.1	3.3
Total *n*	900,441	949,347	803,300	803,300	463,049	463,049	463,049

## Measures

### School burnout

For measuring school burnout, we used the Short School Burnout Inventory (SSBI) [25, 26] which was developed for the purposes of the School Health Promotion Survey [7]. The Short School Burnout Scale has three items measuring (1) exhaustion at school (I feel overwhelmed by schoolwork), (2) lack of the meaning of cynicism toward the meaning of school (I feel of loss of interest in schoolwork), and (3) sense of inadequacy at school (I often have feelings of inadequacy at school). Each item is rated on a 4-point scale (1 = not at all; 4 = daily). The Cronbach’s α reliability for the scale was 0.75 for boys and 0.74 for girls in lower secondary school and 0.71 for boys and 0.73 for girls in upper secondary school (see also [25]).

### Socioeconomic factors

We added socioeconomic variables to the model to measure the resources (parental educational level) and demands (parental unemployment, urban area, and immigration). These factors were known to be associated with burnout as described in the introduction. A dichotomous measure was used for the educational level of the parents: (0) below degree in higher education; (1) one or more parents had a degree in higher education. Urban–rural characteristics of the area of the school were measured using three categories: (1) urban, (2) semi-urban, (3) rural. Parental employment status was measured with whether the parents have been unemployed during the last 12 months: (0) Nonunemployed, (1) One has been unemployed, and (2) Both have been unemployed. Parental employment status was not available for the latest data collection year 2019. Immigration status was measured using four categories: (1) Native, (2) One parent foreign-born, (3) Born in Finland, foreign-born parents, and (4) Born abroad, foreign-born parents. Immigration status was available only for the years 2013–2019.

### Analysis

We first explored the distributions of school burnout by the year of survey, gender, school level, and socioeconomic factors. We used Generalized Linear Models (GLM) in STATA 17 to study the effects of these characteristics on school burnout trends. To assess the effect of time, we included an interaction term which also allowed the variation between the school levels and genders (Model 1). After that, we added the socioeconomic variables (parental education, urban–rural area, parental unemployment, and family immigrations status) to the three-way interaction to see if there are gender and/or school level-specific trends in school burnout over time that depend on the socioeconomic factors. We tested four interactions: year*gender*school level*parental education (Model 2), year*gender*school level*parental employment status (Model 2), year*gender*school level*urban–rural (Model 3), and year*gender*school level*immigration status (Model 4). Urban–rural area and immigrations status were available for a shorter time and therefore tested in different models. We examined the interaction terms to see how the effect of the socioeconomic factors on school burnout differed by year, gender, and/or school level. To determine whether an interaction term was necessary to keep in the model, the Wald test for the interaction term was carried out. A p value smaller than 0.05 was used as an indication of a significant interaction effect. Because of many interaction terms (due to categorical variables), the tables show the results for Wald tests for interactions. The code used and the detailed estimates for the interactions are shown in Online Resource 2. Figures were used to illustrate the key associations, based on the estimated marginal means and confidence intervals calculated from the GLM models.

## Results

### Descriptive results

Online Resource 1 shows the proportions of the participants by the study year. Table 1 shows the distributions of school burnout and sociodemographic factors in boys and girls in lower and upper secondary school. Between 2006 and 2019, the average school burnout was 1.8 (sd = 0.75) and 2.0 (sd = 0.70) in girls and 1.9 (sd = 0.74) and 1.7 (sd = 0.65) in boys in lower and upper secondary schools, respectively. About 66% of the students in lower school and 73% in upper secondary school were living in an urban area. A quarter reported that at least one parent has been unemployed during the last 12 months. About 6–7% of the students had a foreign-born parent. A smaller proportion was born in Finland (2%) or born abroad (2–4%) to foreign-born parents. Missingness was relatively low: less than 1% for school burnout and urban–rural classification, 1–3% for parental employment status, and 1–8% for parental education and immigration status. The proportions varied between the genders and the school levels (see Table 1). Table 2 shows the distributions of the socioeconomic factors by the year of the survey. The proportions of parental higher education degrees and urban residency increased between 2006 and 2019. Parental unemployment increased (available between 2006 and 2017), as well as proportions of students with immigrant background (available between 2013 and 2019).

### The effects of year, school level, gender, and socioeconomic factors on school burnout

Table 3 shows the effects of year, gender, and school level on school burnout (Model 1) adding two of the socioeconomic factors, parental education, and urban–rural, between 2006 and 2019 (Model 2). There was an overall increase (although non-linear) in school burnout from 2006 to 2019, especially after 2011. Higher school burnout was associated with female gender, being in upper secondary school, lower parental education, and living in urban areas. Model 1 shows that there was an interaction between year, gender, and school level. Girls in upper secondary school expressed consistently higher school burnout compared to the girls in lower secondary school (Figure in Online Resource 3). However, the school level made little difference in school burnout among boys, apart from between 2008 and 2013 when school burnout was higher among boys in lower secondary school compared to their counterparts in upper secondary school.

**Table 3:** Generalized linear models (GLM) for school burnout between 2006 and 2019

	School burnout	
	Model 1	Model 2
*Main effects*	*n* = 897,982	*n* = 897,982
Year of survey (ref = 2006–2007)		
2008–2009	0.01*	0.01**
2010–2011	− 0.02***	− 0.02***
2013	− 0.03***	− 0.03***
2015	0.00	0.01*
2017	− 0.03***	− 0.02***
2019	0.03***	0.04***
School level (ref = upper secondary school)	− 0.04***	0.04***
Female	0.20***	0.20***
Parent(s) with HE degree	–	− 0.05***
Urban–rural (ref = urban)		
Semi-urban	–	− 0.02***
Rural	–	− 0.03***
*Wald tests for interactions:*	Wald (df)	Wald (df)
Year*gender*school level*parental education	–	3.36 (6)
Year*gender*school level*urban–rural	–	16.21 (12)
Year*gender*parental education	–	9.51 (6)
Year*gender*urban–rural	–	29.96 (12) **
Year*school level*parental education	–	4.84 (6)
Year*school level*urban–rural	–	15.44 (12)
Year*gender*school level	54.34 (6)***	25.83 (6) ***
Year*gender	2419.36 (6)***	1381 (6) ***
Year*school level	133.10 (6)***	75.88 (6)***
Year*parental education	–	14.3 (6) *
Year* urban–rural	–	46.75 (12)***
Gender*school level*parental education	–	1.98 (1)
Gender*school level*urban–rural	–	12.99 (2) **
Gender*school level	–	159.64 (1) ***
Gender*parental education	–	9.14 (1) **
Gender* urban–rural	–	55.63 (2)***
School level*parental education	–	3.16 (1)
School level*urban–rural	–	2.89 (2)

Unstandardized estimates shown, *df* degrees of freedom

***p < 0.001, **p < 0.01, *p < 0.05

Two four-way interactions were first tested: year*gender*school level*parental education and year*gender*school level*urban–rural (Table 3, Model 2). Neither of them was significant. Parental education appeared to have a similar effect over time and regardless of educational level. The difference between the boys’ school burnout by parental education was slightly larger compared to girls. Figure in Online Resource 4 depicts the year, gender, and parental education (whether any parent had a degree or not) on school burnout.

The interaction between the year, gender, and urban–rural area was significant (Table 3, Model 2). Among boys, the differences in school burnout by the urban, semi-urban, and rural areas were generally very small with a slightly widening difference towards the last years of the study (Figure in Online Resource 5). In girls, school burnout was higher in urban compared to rural areas throughout the years (except for 2013). The school level (lower vs. upper secondary school) was not significant in the interaction related to parental education and urban–rural area.

Table 4 shows the associations between parental employment status and school burnout between 2006 and 2017. An interaction for year*gender*school level*parental employment status was first tested. Similar to the two previous models, school level was not significant in the interaction. The results show that the interaction for year, gender, and parental employment status was significant. Figure in Online Resource 6 depicts this interaction. The level of school burnout among boys with unemployed parents was higher than girls with parents in employment. Especially at the start of the study (2006–2007), boys with unemployed parents showed as high levels of school burnout as their female counterparts. Parental unemployment had a larger effect among the students in lower secondary school compared to the students in upper secondary school across the time (school level*parental unemployment).

**Table 4:** Generalized linear models (GLM) for school burnout between 2006 and 2017

	Model 3
*Main effects*	*n* = 771,564
Year of survey (ref = 2006–2007)	
2008–2009	0.00
2010–2011	− 0.03***
2013	− 0.04***
2015	− 0.00
2017	− 0.03***
School level (ref upper secondary school)	0.04***
Female	0.17***
Parent(s) with HE degree	-0.04***
Urban–rural (ref = urban)	
Semi-urban	− 0.01***
Rural	− 0.02***
Parent(s) unemployed (ref = none)	
One	0.10***
Two or more	0.31***
*Wald tests for interactions:*	Wald (df)
Year*gender*school level*parent(s) unemployed	6.10 (10)
Year*gender*parent(s) unemployed	50.24 (10)***
Year*school level*parent(s) unemployed	8.31 (10)
Year*gender*school level	6.75 (5)
Year*gender	552.54 (5)***
Year*school level	62.43 (5)***
Year*parent(s) unemployed	58.78 (10)***
gender*school level*parent(s) unemployed	0.41 (2)
Gender*school level	193.45 (1)***
Gender*parent(s) unemployed	34.83 (2)***
School level*parent(s) unemployed	15.15 (2)***

Unstandardized estimates shown, *df* degrees of freedom

***p < 0.001, **p < 0.01, *p < 0.05

Table 5 shows the associations between immigration status and school burnout between 2013 and 2019. A four-way interaction for year*gender*school level*immigration status was first tested. This interaction was not significant but the three-way interaction year*gender*immigrations status was significant. Figure in Online Resource 7 shows the interaction for year, gender, and immigrations status: boys generally showed lower school burnout than girls, except for boys born abroad to foreign-born parents. The highest levels of school burnout in girls were among those with one foreign-born parent. The patterns of school burnout by immigrations status were similar across the school levels (none of the interactions involving school level and immigration were significant).

**Table 5:** Generalized linear models (GLM) for school burnout between 2013 and 2019

	Model 4
*Main effects*	*n* = 444,726
Year of survey (ref = 2013)	
2015	− 0.01
2017	− 0.07***
2019	− 0.05***
School level (ref upper secondary school)	− 0.03***
Female	0.16***
Parent(s) with HE degree	− 0.06***
Urban–rural (ref = urban)	
Semi-urban	− 0.02***
Rural	− 0.03***
Immigration status (ref = native)	
One parent foreign-born	0.12***
Foreign-born parents, born in Finland	0.14***
Foreign-born parents, born abroad	0.37***
*Wald tests for interactions:*	Wald (*df*)
Year*gender*school level*immigration status	7.85 (6)
Year*gender* immigration status	17.21 (6)**
Year*school level* immigration status	11.54 (6)
Year*gender*school level	8.45 (2)*
Year*gender	236.12 (2)***
Year*school level	43.75 (2)***
Year* immigration status	27.34 (6)***
gender*school level* immigration status	2.45 (3)
Gender*school level	189.50 (1)***
Gender* immigration status	64.98 (3)***
School level* immigration status	2.95 (3)

Unstandardized estimates shown, *df* degrees of freedom

***p < 0.001, **p < 0.01, *p < 0.05

### The interaction between resource factor (parental education) and the demands factors (parental unemployment, urban area, and immigration)

We added the interaction terms between parental education and parental unemployment, urban area, and immigration to assess the potential buffer effect of parental education. None of the interactions between parental education and urban area were significant. The interaction year*parental education*parental unemployment (Wald test estimate = 49.32, degrees of freedom = 10, *p* < 0.001) showed that a parental higher education degree was associated with a lower school burnout, especially in those families with two or more parents unemployed after 2011 (Figure in Online Resource 8). There was also an interaction between immigration status and parental higher degree (Wald test estimate = 49.32, degrees of freedom = 10, *p* < 0.001), so that the parental degree played the largest buffering role in school burnout among the students who themselves and their parents were born abroad (Figure in Online Resource 9).

## Discussion

Our study is to our knowledge first to investigate longer term trends in school burnout among secondary school students. The results echoed the earlier findings of declining well-being trends in young people in Europe [27, 28] and the United States [29, 30]. They highlight the role of gender and the interplay between the resource and demand factors over time.

### The overall trend of school burnout

The results show as expected that the overall trend of school burnout over the period from 2006 to 2019 increased. However, the pattern suggested a relatively flat trend for girls and even decline among boys in school burnout up to 2011, after which the school burnout started to increase sharply among girls and show some increase among boys. This would coincidence with the educational budget cuts in Finland [23]. It is noteworthy that the models adjust for the personal socioeconomic resources and demands. Thus, the increasing school burnout trend after 2011 happens regardless of the variation in family level education, employment, and urban–rural residence during this period of time.

### The impact of gender and school level on school burnout

As expected, the school burnout trend was gendered, in line with the previous findings [9]. Girls experienced higher and increasing levels of school burnout between 2006 and 2019. Boys, in turn, started with lower levels of school burnout which decreased. There was also a gender difference between the school levels: girls in upper secondary school consistently reported the highest school burnout and higher than girls in lower secondary school. In boys, school burnout was fairly similar in lower and upper secondary school and when it showed any difference (between 2008 and 2013) than in favour of lower school burnout among boys in upper secondary school.

The results of gender and school level may reflect the stage-environment fit theory, positing that students’ motivation and engagement are largely determined by the extent to which secondary schools provide educational and social environments that meet adolescents’ needs for relatedness, autonomy, and competence [31]. These environments may have a different fit depending on gender and school level. From the 7th to the 9th grades, students experience a significant decline in looking forward to school and find school increasingly exhausting [32]. Indeed, Finnish 15 years old fall well below the OECD average in international comparisons of how happy students feel at school [6]. The trend towards motivational declines is the most marked for students who choose the academic track after their transition to upper secondary education, a choice more frequent in girls than boys [33]. Within a year and a half after entering upper secondary school in 10th grade, students who are on the academic track feel significantly burned out [9]. As part of their preparation for university study, students on the academic track strive to be successful with schoolwork that is more challenging and strenuous in an educational environment that is oriented more towards social comparison and competition among peers than it was in their years of basic education. The higher and increasing school burnout among girls may also reflect the gendered coping mechanism in the school environment. Previous research has shown internalizing symptoms being more prevalent among girls compared to boys in these school contexts [34]. This may contribute to higher expressed school burnout levels in girls.

### The impact of socioeconomic resources and demands on school burnout

There were several modifying socioeconomic factors in the school burnout trends. As described above the interplay between gender and school level impacted how school burnout evolved in lower and upper secondary schools between 2006 and 2019. Adding other socioeconomic factors to this year*gender*school level interaction showed that most of the socioeconomic effects on school burnout were gender and time-dependent but differed less by school level. There was no consistent socioeconomic gradient by gender: Urban area was associated with school burnout to an increasing degree among girls, while parental unemployment and lower educational levels were associated with somewhat higher school burnout among boys.

Parental educational level showed a fairly uniform effect over time: lower parental education was associated with higher school burnout regardless of school level and year of the survey. The result is in line with some previous longitudinal findings focusing on lower secondary schools in Finland [16]. The current results also point to an overall gender effect: the difference between the boys’ school burnout by parental education was slightly larger compared to girls regardless of the year of the study.

Urban–rural areas had more impact towards the end of the study period: girls in urban areas had increased school burnout, whereas among boys, the area of residence made very little difference in school burnout. Again, the results suggested a gendered pattern of school burnout. Urban segregation in school choice and increased competition to selective schools, most located in urban areas, have been identified for the recent years in Finland [35]. This trend may especially affect girls who are more likely to choose and be chosen to selective schools as they often academically outperform boys [36].

Parental unemployment was associated with higher school burnout. Boys with more than one parent unemployed showed especially high levels of school burnout at the start of the study: the usual gender gradient (a higher school burnout among girls compared to boys) disappeared and these boys’ school burnout scores were similar to their female counterparts. The results also showed that parental unemployment had a larger effect among students in lower secondary school compared to the students in upper secondary school which was similar across the time. Parental education appeared to play an increasing buffering impact on school burnout among those students with parents unemployed after 2011.

Immigration status also showed a gendered pattern that changed over time. Although boys generally showed lower school burnout than girls, boys born abroad to foreign-born parents had the highest school burnout in 2013 and 2015. The highest levels of school burnout in girls were among those with one foreign-born parent. The patterns of school burnout by immigrations status were similar across the school levels. Previous research has found that recently immigrated boys have especially high levels of school burnout [16]. The results point to the importance of gender and time of immigration in school burnout. Parental degrees showed an important buffering role in school burnout, especially among the students who themselves and their parents were born abroad.

### Limitations

The trends in the study are based on cross-sectional data. Therefore, little can be said about the individual change of school burnout over time. As this is an observational study, it is not possible to separate the effects of different policy changes. For this, we would require a control group of students experiencing different policy contexts. The effects of policy changes may not be immediate. The study covered the trends for 5 years before and 7 years after the school budget cuts. However, it is possible that the effects may take even a longer time. The data also did not provide other contextual factors on the schools which may be relevant considering the relative independence of schools to make local decisions in Finland [23]. There was some non-response (e.g., those absent from school or not able to participate because of disability or weak language skills). The family socioeconomic data were self-reported by the student and may contain inaccuracies. We did not have a full time period covered for parental unemployment and family immigration status.

Most data were self-reports in a questionnaire, which may induce common method bias within the cross-sectional measurements (e.g., answering style using middle or extreme ends of the scale, answers impacted by concurrent mood, or social desirability). The common method bias is less likely to affect the relationship of socioeconomic demands/resources and school burnout due to their methodological and psychological distance as different types of measures. The demands/resources assessed categorical sociodemographic conditions, whereas school burnout was the only trait-like characteristic, see [37]. Despite good internal consistency according to Cronbach’s alpha, the composite score for school burnout may contain measurement error.

Despite these limitations, the current study provides a unique opportunity to understand trends in school burnout and socioeconomic factors in a highly representative sample of secondary school students in Finland. The long time period allows the exploration of trends over thirteen years from 2006, while many substantial changes in socioeconomic resources and demands and school policies have taken place. Although the school context in Finland is unique and the results may not be easily generalized to other countries, the general trends of socioeconomic factors are similar to what many countries in Europe have experienced. The illustration of the interplay between socioeconomic factors and school burnout trends might be useful in other contexts. This information is valuable in enhancing well-being at school and identifying those groups that need additional attention.

### Conclusions

The results showed that the trends in school burnout are often gendered. The underlying general pattern of increasing school burnout among girls and a slight decline of burnout among boys is however interrupted by socioeconomic factors. Educational level of the parents seems to have a constant impact over time; the gradient for boys slightly larger compared to girls. Urban area contributed to the trend of increasing school burnout among girls but not among boys. In addition to considering the higher risk of school burnout related to lower parental education and urbanization, it is important to pay attention to support those students whose parents have no higher degree and no job or with immigration background, especially boys born abroad with foreign-born parents and girls with one foreign-born parent. Although parental education appears to mitigate the child’s school burnout when the family experiences unemployment or immigration, these groups seem to express especially high levels with an often increasing trend of school burnout over time. Moreover, the education budget cuts from 2011 onwards may have contributed to the trend of increasing school burnout among girls and stagnation of school burnout decline among boys. Although local decision-makers may argue that schools in Finland have fared well with a reduced education budget [38], the current study demonstrates that the sustained results may have been achieved at the expense of students’ well-being.

## Supplementary Information

Below is the link to the electronic supplementary material.

Supplementary file1 (DOCX 14 KB)

Supplementary file2 (DOCX 61 KB)

Supplementary file3 (DOCX 1227 KB)

## Data Availability

The data that support the findings of this study are available from The Finnish Institute for Health and Welfare, but restrictions apply to the availability of these data, which were used under license for the current study, and so are not publicly available. Data are, however, available from the authors upon reasonable request and with permission of the Finnish Institute for Health and Welfare.

## Code availability

The Stata code used for the analysis and the full results are in Online Resource 2.
