# Supplementary material for: School burnout trends and sociodemographic factors in Finland 2006–2019
Source: Soc Psychiatry Psychiatr Epidemiol. 2022 Mar 22;57(8):1659–69. doi: 10.1007/s00127-022-02268-0 (PMC9288953; doi:10.1007/s00127-022-02268-0)
Supplement: Supplementary file 1 — Supplementary file1 (DOCX 14 KB) [file 127_2022_2268_MOESM1_ESM.docx]

Online Resource 1. Distributions of the girls and boys in lower and upper secondary school in the School Health Promotion Study 2006-2019.

|  | Girls - Lower | | Girls - Upper | | | Boys - Lower | | Boys - Upper | |  |
| --- | --- | --- | --- | --- | --- | --- | --- | --- | --- | --- |
|  | *n* | | *n* | | | *n* | | *n* | |  |
|  | 315,174 |  | | 184,148 |  | | 315,214 |  | 133,717 |  |
| Year of survey |  |  | |  |  | |  |  |  |  |
| 2006-07 | 54,286 |  | | 30,209 |  | | 54,841 |  | 22,553 |  |
| 2008-09 | 54,216 |  | | 29,286 |  | | 54,433 |  | 21,181 |  |
| 2010-11 | 51,216 |  | | 27,795 |  | | 51,329 |  | 20,588 |  |
| 2013 | 49,255 |  | | 27,771 |  | | 50,223 |  | 20,839 |  |
| 2015 | 25,257 |  | | 22,784 |  | | 25,147 |  | 15,976 |  |
| 2017 | 36,883 |  | | 20,062 |  | | 36,276 |  | 14,323 |  |
| 2019 | 44,061 |  | | 26,241 |  | | 42,965 |  | 18,257 |  |
