# Supplementary material for: School burnout trends and sociodemographic factors in Finland 2006–2019
Source: Soc Psychiatry Psychiatr Epidemiol. 2022 Mar 22;57(8):1659–69. doi: 10.1007/s00127-022-02268-0 (PMC9288953; doi:10.1007/s00127-022-02268-0)
Supplement: Supplementary file 2 — Supplementary file2 (DOCX 61 KB) [file 127_2022_2268_MOESM2_ESM.docx]

. glm burnout i.year i.school_level i.gender i.education i.urbanrural,family(gaussian) li

> nk(identity)

Iteration 0: log likelihood = -976852.54

Generalized linear models No. of obs = 897,982

Optimization : ML Residual df = 897,970

Scale parameter = .5157145

Deviance = 463096.1448 (1/df) Deviance = .5157145

Pearson = 463096.1448 (1/df) Pearson = .5157145

Variance function: V(u) = 1 [Gaussian]

Link function : g(u) = u [Identity]

AIC = 2.175688

Log likelihood = -976852.5365 BIC = -1.18e+07

----------------------------------------------------------------------------------------

| OIM

burnout | Coef. Std. Err. z P>|z| [95% Conf. Interval]

-----------------------+----------------------------------------------------------------

year |

2008-2009 | .0082505 .002601 3.17 0.002 .0031526 .0133485

2010-2011 | -.0168358 .0026285 -6.41 0.000 -.0219875 -.0116841

2013 | -.0291118 .0026661 -10.92 0.000 -.0343373 -.0238863

2015 | .0078362 .0030607 2.56 0.010 .0018374 .013835

2017 | -.0177846 .0029314 -6.07 0.000 -.02353 -.0120392

2019 | .0418914 .0027568 15.20 0.000 .0364882 .0472946

|

school_level |

Upper | .041273 .0016154 25.55 0.000 .0381069 .0444392

|

gender |

Girl | .2014804 .0015242 132.19 0.000 .198493 .2044678

1.education | -.0530419 .0015657 -33.88 0.000 -.0561107 -.0499731

|

urbanrural |

Semiurban| -.0184468 .0020755 -8.89 0.000 -.0225146 -.0143789

Rural | -.025224 .0021952 -11.49 0.000 -.0295265 -.0209214

|

_cons | 1.786857 .0022358 799.21 0.000 1.782475 1.791239

----------------------------------------------------------------------------------------

. glm burnout i.year##i.school_level##i.gender##i.urbanrural i.education,family(gaussian)

> link(identity)

Iteration 0: log likelihood = -974388.14

Generalized linear models No. of obs = 897,982

Optimization : ML Residual df = 897,897

Scale parameter = .5129333

Deviance = 460561.2924 (1/df) Deviance = .5129333

Pearson = 460561.2924 (1/df) Pearson = .5129333

Variance function: V(u) = 1 [Gaussian]

Link function : g(u) = u [Identity]

AIC = 2.170362

Log likelihood = -974388.1407 BIC = -1.18e+07

-----------------------------------------------------------------------------------------

| OIM

burnout | Coef. Std. Err. z P>|z| [95% Conf. Interval]

------------------------+----------------------------------------------------------------

year |

2008-2009 | .0127981 .0056098 2.28 0.023 .0018031 .0237931

2010-2011 | -.009214 .005653 -1.63 0.103 -.0202938 .0018658

2013 | -.0441012 .0057456 -7.68 0.000 -.0553623 -.0328401

2015 | -.0493378 .0069504 -7.10 0.000 -.0629605 -.0357152

2017 | -.1067048 .0064155 -16.63 0.000 -.1192789 -.0941307

2019 | -.0836811 .0059729 -14.01 0.000 -.0953879 -.0719744

|

school_level |

Upper | -.005554 .0069616 -0.80 0.425 -.0191983 .0080904

|

year#school_level |

2008-2009#Upper | -.0206904 .0099192 -2.09 0.037 -.0401317 -.0012492

2010-2011#Upper | -.051927 .0099258 -5.23 0.000 -.0713812 -.0324728

2013#Upper | -.0448161 .0099623 -4.50 0.000 -.0643419 -.0252903

2015#Upper | .0159909 .0111941 1.43 0.153 -.0059492 .037931

2017#Upper | .023053 .0111843 2.06 0.039 .0011322 .0449738

2019#Upper | .0305612 .0103435 2.95 0.003 .0102884 .0508341

|

gender |

Girl | .0988454 .0056194 17.59 0.000 .0878316 .1098592

|

year#gender |

2008-2009#Girl | .0129718 .0079123 1.64 0.101 -.0025359 .0284796

2010-2011#Girl | .0158051 .0079808 1.98 0.048 .0001631 .0314472

2013#Girl | .0504278 .008102 6.22 0.000 .0345483 .0663074

2015#Girl | .1042529 .009755 10.69 0.000 .0851336 .1233723

2017#Girl | .1851325 .008932 20.73 0.000 .167626 .2026389

2019#Girl | .2639482 .0083327 31.68 0.000 .2476165 .28028

|

school_level#gender |

Upper#Girl | .1009389 .0094057 10.73 0.000 .0825041 .1193738

|

year#school_level#|

gender |

2008-2009#Upper#Girl | .0074599 .0133593 0.56 0.577 -.0187239 .0336437

2010-2011#Upper#Girl | .0232101 .0134143 1.73 0.084 -.0030815 .0495017

2013#Upper#Girl | .0301769 .0134696 2.24 0.025 .0037769 .0565768

2015#Upper#Girl | -.0053925 .0150734 -0.36 0.721 -.0349358 .0241508

2017#Upper#Girl | -.0243357 .0149643 -1.63 0.104 -.0536653 .0049939

2019#Upper#Girl | -.0573779 .0138405 -4.15 0.000 -.0845049 -.030251

|

urbanrural |

Semiurban| .0203258 .0083675 2.43 0.015 .0039258 .0367258

Rural | .0300563 .0084165 3.57 0.000 .0135603 .0465524

|

year#urbanrural |

2008-2009 #|

Semiurban| -.0284522 .0119433 -2.38 0.017 -.0518607 -.0050437

2008-2009 #|

Rural | -.0217824 .0121044 -1.80 0.072 -.0455066 .0019418

2010-2011 #|

Semiurban| -.0111484 .0120588 -0.92 0.355 -.0347832 .0124865

2010-2011 #|

Rural | -.0365134 .0123229 -2.96 0.003 -.0606658 -.012361

2013 #|

Semiurban| -.0262257 .0122955 -2.13 0.033 -.0503245 -.0021269

2013 #|

Rural | -.035771 .0126011 -2.84 0.005 -.0604687 -.0110732

2015 #|

Semiurban| -.024074 .0149793 -1.61 0.108 -.0534328 .0052848

2015 #|

Rural | -.027737 .0156212 -1.78 0.076 -.058354 .00288

2017 #|

Semiurban| -.0462912 .0134988 -3.43 0.001 -.0727484 -.019834

2017 #|

Rural | -.0386306 .013904 -2.78 0.005 -.065882 -.0113792

2019 #|

Semiurban| -.0311509 .0127656 -2.44 0.015 -.056171 -.0061308

2019 #|

Rural | -.0775592 .0134917 -5.75 0.000 -.1040024 -.051116

|

school_level#urbanrural |

Upper #|

Semiurban| .0012978 .015882 0.08 0.935 -.0298304 .0324259

Upper #|

Rural | -.0275474 .0166542 -1.65 0.098 -.060189 .0050943

|

year#school_level#|

urbanrural |

2008-2009 #|

Upper #|

Semiurban| -.0307348 .0229497 -1.34 0.180 -.0757155 .0142458

2008-2009 #|

Upper #|

Rural | .0193853 .0239068 0.81 0.417 -.0274712 .0662419

2010-2011 #|

Upper #|

Semiurban| -.0090244 .0231928 -0.39 0.697 -.0544815 .0364327

2010-2011 #|

Upper #|

Rural | .026732 .0248385 1.08 0.282 -.0219506 .0754146

2013 #|

Upper #|

Semiurban| -.0022234 .0233665 -0.10 0.924 -.048021 .0435742

2013 #|

Upper #|

Rural | .0776032 .0252063 3.08 0.002 .0281997 .1270066

2015 #|

Upper #|

Semiurban| -.0239261 .0257296 -0.93 0.352 -.0743553 .0265031

2015 #|

Upper #|

Rural | .0162467 .0278155 0.58 0.559 -.0382706 .070764

2017 #|

Upper #|

Semiurban| -.014246 .0255377 -0.56 0.577 -.0642989 .0358069

2017 #|

Upper #|

Rural | .0082136 .027581 0.30 0.766 -.0458441 .0622713

2019 #|

Upper #|

Semiurban| -.039768 .0240021 -1.66 0.098 -.0868113 .0072753

2019 #|

Upper #|

Rural | .0216415 .0265214 0.82 0.415 -.0303396 .0736225

|

gender#urbanrural |

Girl #|

Semiurban| -.0567128 .0118762 -4.78 0.000 -.0799897 -.0334359

Girl #|

Rural | -.0791137 .0118938 -6.65 0.000 -.1024252 -.0558022

|

year#gender#|

urbanrural |

2008-2009 #|

Girl #|

Semiurban| .028353 .0169211 1.68 0.094 -.0048118 .0615179

2008-2009 #|

Girl #|

Rural | .0187871 .0170804 1.10 0.271 -.01469 .0522641

2010-2011 #|

Girl #|

Semiurban| .0312466 .0170806 1.83 0.067 -.0022308 .0647239

2010-2011 #|

Girl #|

Rural | .0506094 .0173891 2.91 0.004 .0165273 .0846915

2013 #|

Girl #|

Semiurban| .0523351 .0174087 3.01 0.003 .0182146 .0864556

2013 #|

Girl #|

Rural | .0611697 .0177961 3.44 0.001 .02629 .0960493

2015 #|

Girl #|

Semiurban| .0340653 .0211569 1.61 0.107 -.0074014 .0755321

2015 #|

Girl #|

Rural | .0263287 .0220513 1.19 0.232 -.0168911 .0695485

2017 #|

Girl #|

Semiurban| .0421177 .0188934 2.23 0.026 .0050873 .0791481

2017 #|

Girl #|

Rural | -.0028847 .0194765 -0.15 0.882 -.041058 .0352886

2019 #|

Girl #|

Semiurban| .0053888 .0179041 0.30 0.763 -.0297026 .0404801

2019 #|

Girl #|

Rural | .0213155 .0189188 1.13 0.260 -.0157647 .0583958

|

school_level#gender#|

urbanrural |

Upper #|

Girl #|

Semiurban| .0456217 .0214399 2.13 0.033 .0036003 .0876431

Upper #|

Girl #|

Rural | .0725956 .0223187 3.25 0.001 .0288518 .1163393

|

year#school_level#|

gender#urbanrural |

2008-2009 #|

Upper #|

Girl #|

Semiurban| -.004954 .0308824 -0.16 0.873 -.0654825 .0555744

2008-2009 #|

Upper #|

Girl #|

Rural | -.022048 .0320471 -0.69 0.491 -.0848591 .0407631

2010-2011 #|

Upper #|

Girl #|

Semiurban| -.0405891 .0312158 -1.30 0.194 -.101771 .0205929

2010-2011 #|

Upper #|

Girl #|

Rural | -.063216 .0331961 -1.90 0.057 -.1282792 .0018472

2013 #|

Upper #|

Girl #|

Semiurban| -.0578395 .0316059 -1.83 0.067 -.119786 .004107

2013 #|

Upper #|

Girl #|

Rural | -.1029787 .0336197 -3.06 0.002 -.1688722 -.0370852

2015 #|

Upper #|

Girl #|

Semiurban| -.0029674 .0347345 -0.09 0.932 -.0710457 .0651109

2015 #|

Upper #|

Girl #|

Rural | -.0420575 .0373049 -1.13 0.260 -.1151738 .0310589

2017 #|

Upper #|

Girl #|

Semiurban| -.0444345 .0342259 -1.30 0.194 -.1115161 .022647

2017 #|

Upper #|

Girl #|

Rural | -.0341694 .0366638 -0.93 0.351 -.1060291 .0376903

2019 #|

Upper #|

Girl #|

Semiurban| -.0044534 .0321887 -0.14 0.890 -.0675421 .0586353

2019 #|

Upper #|

Girl #|

Rural | -.0154143 .0351465 -0.44 0.661 -.0843003 .0534716

|

1.education | -.0528124 .0015624 -33.80 0.000 -.0558747 -.0497502

_cons | 1.830889 .0040381 453.40 0.000 1.822975 1.838804

-----------------------------------------------------------------------------------------

. testparm year#school_level#gender#urbanrural

( 1) [burnout]7.year#20.school_level#2.gender#2.urbanrural = 0

( 2) [burnout]7.year#20.school_level#2.gender#3.urbanrural = 0

( 3) [burnout]8.year#20.school_level#2.gender#2.urbanrural = 0

( 4) [burnout]8.year#20.school_level#2.gender#3.urbanrural = 0

( 5) [burnout]9.year#20.school_level#2.gender#2.urbanrural = 0

( 6) [burnout]9.year#20.school_level#2.gender#3.urbanrural = 0

( 7) [burnout]10.year#20.school_level#2.gender#2.urbanrural = 0

( 8) [burnout]10.year#20.school_level#2.gender#3.urbanrural = 0

( 9) [burnout]11.year#20.school_level#2.gender#2.urbanrural = 0

(10) [burnout]11.year#20.school_level#2.gender#3.urbanrural = 0

(11) [burnout]12.year#20.school_level#2.gender#2.urbanrural = 0

(12) [burnout]12.year#20.school_level#2.gender#3.urbanrural = 0

chi2( 12) = 16.21

Prob > chi2 = 0.1820

. testparm year#gender#education

no such variables;

the specified varlist does not identify any testable coefficients

r(111);

. testparm year#gender#urbanrural

( 1) [burnout]7.year#2.gender#2.urbanrural = 0

( 2) [burnout]7.year#2.gender#3.urbanrural = 0

( 3) [burnout]8.year#2.gender#2.urbanrural = 0

( 4) [burnout]8.year#2.gender#3.urbanrural = 0

( 5) [burnout]9.year#2.gender#2.urbanrural = 0

( 6) [burnout]9.year#2.gender#3.urbanrural = 0

( 7) [burnout]10.year#2.gender#2.urbanrural = 0

( 8) [burnout]10.year#2.gender#3.urbanrural = 0

( 9) [burnout]11.year#2.gender#2.urbanrural = 0

(10) [burnout]11.year#2.gender#3.urbanrural = 0

(11) [burnout]12.year#2.gender#2.urbanrural = 0

(12) [burnout]12.year#2.gender#3.urbanrural = 0

chi2( 12) = 29.96

Prob > chi2 = 0.0028

. testparm year#school_level#urbanrural

( 1) [burnout]7.year#20.school_level#2.urbanrural = 0

( 2) [burnout]7.year#20.school_level#3.urbanrural = 0

( 3) [burnout]8.year#20.school_level#2.urbanrural = 0

( 4) [burnout]8.year#20.school_level#3.urbanrural = 0

( 5) [burnout]9.year#20.school_level#2.urbanrural = 0

( 6) [burnout]9.year#20.school_level#3.urbanrural = 0

( 7) [burnout]10.year#20.school_level#2.urbanrural = 0

( 8) [burnout]10.year#20.school_level#3.urbanrural = 0

( 9) [burnout]11.year#20.school_level#2.urbanrural = 0

(10) [burnout]11.year#20.school_level#3.urbanrural = 0

(11) [burnout]12.year#20.school_level#2.urbanrural = 0

(12) [burnout]12.year#20.school_level#3.urbanrural = 0

chi2( 12) = 15.44

Prob > chi2 = 0.2183

. testparm year#urbanrural

( 1) [burnout]7.year#2.urbanrural = 0

( 2) [burnout]7.year#3.urbanrural = 0

( 3) [burnout]8.year#2.urbanrural = 0

( 4) [burnout]8.year#3.urbanrural = 0

( 5) [burnout]9.year#2.urbanrural = 0

( 6) [burnout]9.year#3.urbanrural = 0

( 7) [burnout]10.year#2.urbanrural = 0

( 8) [burnout]10.year#3.urbanrural = 0

( 9) [burnout]11.year#2.urbanrural = 0

(10) [burnout]11.year#3.urbanrural = 0

(11) [burnout]12.year#2.urbanrural = 0

(12) [burnout]12.year#3.urbanrural = 0

chi2( 12) = 46.75

Prob > chi2 = 0.0000

. testparm gender#urbanrural

( 1) [burnout]2.gender#2.urbanrural = 0

( 2) [burnout]2.gender#3.urbanrural = 0

chi2( 2) = 55.63

Prob > chi2 = 0.0000

. testparm school_level#urbanrural

( 1) [burnout]20.school_level#2.urbanrural = 0

( 2) [burnout]20.school_level#3.urbanrural = 0

chi2( 2) = 2.89

Prob > chi2 = 0.2359

. glm burnout i.year##i.school_level##i.gender##i.education i.urbanrural,family(gaussian)

> link(identity)

Iteration 0: log likelihood = -974515.85

Generalized linear models No. of obs = 897,982

Optimization : ML Residual df = 897,924

Scale parameter = .5130638

Deviance = 460692.3097 (1/df) Deviance = .5130638

Pearson = 460692.3097 (1/df) Pearson = .5130638

Variance function: V(u) = 1 [Gaussian]

Link function : g(u) = u [Identity]

AIC = 2.170587

Log likelihood = -974515.8484 BIC = -1.18e+07

------------------------------------------------------------------------------------------

| OIM

burnout | Coef. Std. Err. z P>|z| [95% Conf. Interval]

-------------------------+----------------------------------------------------------------

year |

2008-2009 | -.0051359 .0057295 -0.90 0.370 -.0163656 .0060937

2010-2011 | -.0283784 .0057887 -4.90 0.000 -.039724 -.0170328

2013 | -.0548741 .0060611 -9.05 0.000 -.0667537 -.0429946

2015 | -.0619885 .0074067 -8.37 0.000 -.0765054 -.0474717

2017 | -.1308817 .0069058 -18.95 0.000 -.1444168 -.1173467

2019 | -.1083129 .0066726 -16.23 0.000 -.121391 -.0952348

|

school_level |

Upper | -.0208716 .0079146 -2.64 0.008 -.0363839 -.0053593

|

year#school_level |

2008-2009#Upper | -.0206257 .0116358 -1.77 0.076 -.0434314 .00218

2010-2011#Upper | -.0509848 .0116448 -4.38 0.000 -.0738082 -.0281614

2013#Upper | -.023536 .0123925 -1.90 0.058 -.0478248 .0007529

2015#Upper | .0242986 .013792 1.76 0.078 -.0027332 .0513304

2017#Upper | .0383044 .0143284 2.67 0.008 .0102212 .0663876

2019#Upper | .0454916 .0138991 3.27 0.001 .0182499 .0727334

|

gender |

Girl | .0639564 .0055421 11.54 0.000 .0530941 .0748187

|

year#gender |

2008-2009#Girl | .031392 .0079748 3.94 0.000 .0157618 .0470223

2010-2011#Girl | .0391512 .0080641 4.85 0.000 .0233458 .0549565

2013#Girl | .0814999 .0084788 9.61 0.000 .0648817 .0981181

2015#Girl | .1288875 .0103573 12.44 0.000 .1085875 .1491875

2017#Girl | .2075704 .0095531 21.73 0.000 .1888467 .2262941

2019#Girl | .2848148 .0092639 30.74 0.000 .2666579 .3029716

|

school_level#gender |

Upper#Girl | .130943 .0103637 12.63 0.000 .1106306 .1512554

|

year#school_level#|

gender |

2008-2009#Upper#Girl | -.0035719 .0151526 -0.24 0.814 -.0332705 .0261266

2010-2011#Upper#Girl | .0011165 .0152476 0.07 0.942 -.0287683 .0310012

2013#Upper#Girl | -.01383 .016248 -0.85 0.395 -.0456755 .0180156

2015#Upper#Girl | -.0269028 .0180547 -1.49 0.136 -.0622894 .0084838

2017#Upper#Girl | -.0472557 .0185528 -2.55 0.011 -.0836186 -.0108928

2019#Upper#Girl | -.072822 .0178879 -4.07 0.000 -.1078817 -.0377623

|

1.education | -.0709933 .0065836 -10.78 0.000 -.083897 -.0580897

|

year#education |

2008-2009#1 | .021939 .0092527 2.37 0.018 .003804 .040074

2010-2011#1 | .0262042 .0093467 2.80 0.005 .0078849 .0445235

2013#1 | .0011131 .0094346 0.12 0.906 -.0173784 .0196046

2015#1 | .0082275 .0114479 0.72 0.472 -.0142099 .0306649

2017#1 | .0215075 .010416 2.06 0.039 .0010924 .0419225

2019#1 | .0160412 .0098354 1.63 0.103 -.0032358 .0353182

|

school_level#education |

Upper#1 | .0207291 .0116622 1.78 0.075 -.0021283 .0435866

|

year#school_level#|

education |

2008-2009#Upper#1 | -.0068123 .0166865 -0.41 0.683 -.0395172 .0258926

2010-2011#Upper#1 | -.0020103 .0167943 -0.12 0.905 -.0349266 .0309059

2013#Upper#1 | -.0193491 .0170715 -1.13 0.257 -.0528087 .0141104

2015#Upper#1 | -.0164488 .0190671 -0.86 0.388 -.0538196 .0209219

2017#Upper#1 | -.0289078 .0191819 -1.51 0.132 -.0665035 .008688

2019#Upper#1 | -.0276765 .0182128 -1.52 0.129 -.0633728 .0080199

|

gender#education |

Girl#1 | .0284686 .0094147 3.02 0.002 .0100162 .046921

|

year#gender#education |

2008-2009#Girl#1 | -.0247673 .0132106 -1.87 0.061 -.0506596 .0011251

2010-2011#Girl#1 | -.0212988 .0133476 -1.60 0.111 -.0474597 .004862

2013#Girl#1 | -.0286197 .0134142 -2.13 0.033 -.054911 -.0023283

2015#Girl#1 | -.0326471 .016186 -2.02 0.044 -.0643711 -.0009231

2017#Girl#1 | -.0359891 .0146252 -2.46 0.014 -.0646539 -.0073243

2019#Girl#1 | -.0346751 .0138401 -2.51 0.012 -.0618012 -.007549

|

school_level#gender#|

education |

Upper#Girl#1 | -.0223663 .0158779 -1.41 0.159 -.0534865 .0087538

|

year#school_level#|

gender#education |

2008-2009#Upper#Girl#1 | .0176146 .0225732 0.78 0.435 -.0266281 .0618573

2010-2011#Upper#Girl#1 | .01597 .0227715 0.70 0.483 -.0286612 .0606013

2013#Upper#Girl#1 | .0390784 .0230385 1.70 0.090 -.0060763 .084233

2015#Upper#Girl#1 | .0297424 .0256505 1.16 0.246 -.0205318 .0800165

2017#Upper#Girl#1 | .0262413 .0255492 1.03 0.304 -.0238341 .0763168

2019#Upper#Girl#1 | .0246071 .0241568 1.02 0.308 -.0227393 .0719535

|

urbanrural |

Semiurban| -.018291 .0020702 -8.84 0.000 -.0223486 -.0142334

Rural | -.0252433 .0021898 -11.53 0.000 -.0295353 -.0209514

|

_cons | 1.854793 .0040367 459.48 0.000 1.846882 1.862705

------------------------------------------------------------------------------------------

. margins gender#education

Predictive margins Number of obs = 897,982

Model VCE : OIM

Expression : Predicted mean burnout, predict()

----------------------------------------------------------------------------------

| Delta-method

| Margin Std. Err. z P>|z| [95% Conf. Interval]

-----------------+----------------------------------------------------------------

gender#education |

Poika#0 | 1.791007 .0016143 1109.45 0.000 1.787843 1.794171

Poika#1 | 1.736056 .0016035 1082.65 0.000 1.732913 1.739199

Girl#0 | 1.991998 .0014251 1397.75 0.000 1.989205 1.994792

Girl#1 | 1.941162 .0015848 1224.86 0.000 1.938055 1.944268

----------------------------------------------------------------------------------

. marginsplot

Variables that uniquely identify margins: gender education

. testparm school_level#gender#education

( 1) [burnout]20.school_level#2.gender#1.education = 0

chi2( 1) = 1.98

Prob > chi2 = 0.1589

. glm burnout i.year##i.school_level##i.gender##i.urbanrural i.education,family(gaussian)

> link(identity)

Iteration 0: log likelihood = -974388.14

Generalized linear models No. of obs = 897,982

Optimization : ML Residual df = 897,897

Scale parameter = .5129333

Deviance = 460561.2924 (1/df) Deviance = .5129333

Pearson = 460561.2924 (1/df) Pearson = .5129333

Variance function: V(u) = 1 [Gaussian]

Link function : g(u) = u [Identity]

AIC = 2.170362

Log likelihood = -974388.1407 BIC = -1.18e+07

-----------------------------------------------------------------------------------------

| OIM

burnout | Coef. Std. Err. z P>|z| [95% Conf. Interval]

------------------------+----------------------------------------------------------------

year |

2008-2009 | .0127981 .0056098 2.28 0.023 .0018031 .0237931

2010-2011 | -.009214 .005653 -1.63 0.103 -.0202938 .0018658

2013 | -.0441012 .0057456 -7.68 0.000 -.0553623 -.0328401

2015 | -.0493378 .0069504 -7.10 0.000 -.0629605 -.0357152

2017 | -.1067048 .0064155 -16.63 0.000 -.1192789 -.0941307

2019 | -.0836811 .0059729 -14.01 0.000 -.0953879 -.0719744

|

school_level |

Upper | -.005554 .0069616 -0.80 0.425 -.0191983 .0080904

|

year#school_level |

2008-2009#Upper | -.0206904 .0099192 -2.09 0.037 -.0401317 -.0012492

2010-2011#Upper | -.051927 .0099258 -5.23 0.000 -.0713812 -.0324728

2013#Upper | -.0448161 .0099623 -4.50 0.000 -.0643419 -.0252903

2015#Upper | .0159909 .0111941 1.43 0.153 -.0059492 .037931

2017#Upper | .023053 .0111843 2.06 0.039 .0011322 .0449738

2019#Upper | .0305612 .0103435 2.95 0.003 .0102884 .0508341

|

gender |

Girl | .0988454 .0056194 17.59 0.000 .0878316 .1098592

|

year#gender |

2008-2009#Girl | .0129718 .0079123 1.64 0.101 -.0025359 .0284796

2010-2011#Girl | .0158051 .0079808 1.98 0.048 .0001631 .0314472

2013#Girl | .0504278 .008102 6.22 0.000 .0345483 .0663074

2015#Girl | .1042529 .009755 10.69 0.000 .0851336 .1233723

2017#Girl | .1851325 .008932 20.73 0.000 .167626 .2026389

2019#Girl | .2639482 .0083327 31.68 0.000 .2476165 .28028

|

school_level#gender |

Upper#Girl | .1009389 .0094057 10.73 0.000 .0825041 .1193738

|

year#school_level#|

gender |

2008-2009#Upper#Girl | .0074599 .0133593 0.56 0.577 -.0187239 .0336437

2010-2011#Upper#Girl | .0232101 .0134143 1.73 0.084 -.0030815 .0495017

2013#Upper#Girl | .0301769 .0134696 2.24 0.025 .0037769 .0565768

2015#Upper#Girl | -.0053925 .0150734 -0.36 0.721 -.0349358 .0241508

2017#Upper#Girl | -.0243357 .0149643 -1.63 0.104 -.0536653 .0049939

2019#Upper#Girl | -.0573779 .0138405 -4.15 0.000 -.0845049 -.030251

|

urbanrural |

Semiurban| .0203258 .0083675 2.43 0.015 .0039258 .0367258

Rural | .0300563 .0084165 3.57 0.000 .0135603 .0465524

|

year#urbanrural |

2008-2009 #|

Semiurban| -.0284522 .0119433 -2.38 0.017 -.0518607 -.0050437

2008-2009 #|

Rural | -.0217824 .0121044 -1.80 0.072 -.0455066 .0019418

2010-2011 #|

Semiurban| -.0111484 .0120588 -0.92 0.355 -.0347832 .0124865

2010-2011 #|

Rural | -.0365134 .0123229 -2.96 0.003 -.0606658 -.012361

2013 #|

Semiurban| -.0262257 .0122955 -2.13 0.033 -.0503245 -.0021269

2013 #|

Rural | -.035771 .0126011 -2.84 0.005 -.0604687 -.0110732

2015 #|

Semiurban| -.024074 .0149793 -1.61 0.108 -.0534328 .0052848

2015 #|

Rural | -.027737 .0156212 -1.78 0.076 -.058354 .00288

2017 #|

Semiurban| -.0462912 .0134988 -3.43 0.001 -.0727484 -.019834

2017 #|

Rural | -.0386306 .013904 -2.78 0.005 -.065882 -.0113792

2019 #|

Semiurban| -.0311509 .0127656 -2.44 0.015 -.056171 -.0061308

2019 #|

Rural | -.0775592 .0134917 -5.75 0.000 -.1040024 -.051116

|

school_level#urbanrural |

Upper #|

Semiurban| .0012978 .015882 0.08 0.935 -.0298304 .0324259

Upper #|

Rural | -.0275474 .0166542 -1.65 0.098 -.060189 .0050943

|

year#school_level#|

urbanrural |

2008-2009 #|

Upper #|

Semiurban| -.0307348 .0229497 -1.34 0.180 -.0757155 .0142458

2008-2009 #|

Upper #|

Rural | .0193853 .0239068 0.81 0.417 -.0274712 .0662419

2010-2011 #|

Upper #|

Semiurban| -.0090244 .0231928 -0.39 0.697 -.0544815 .0364327

2010-2011 #|

Upper #|

Rural | .026732 .0248385 1.08 0.282 -.0219506 .0754146

2013 #|

Upper #|

Semiurban| -.0022234 .0233665 -0.10 0.924 -.048021 .0435742

2013 #|

Upper #|

Rural | .0776032 .0252063 3.08 0.002 .0281997 .1270066

2015 #|

Upper #|

Semiurban| -.0239261 .0257296 -0.93 0.352 -.0743553 .0265031

2015 #|

Upper #|

Rural | .0162467 .0278155 0.58 0.559 -.0382706 .070764

2017 #|

Upper #|

Semiurban| -.014246 .0255377 -0.56 0.577 -.0642989 .0358069

2017 #|

Upper #|

Rural | .0082136 .027581 0.30 0.766 -.0458441 .0622713

2019 #|

Upper #|

Semiurban| -.039768 .0240021 -1.66 0.098 -.0868113 .0072753

2019 #|

Upper #|

Rural | .0216415 .0265214 0.82 0.415 -.0303396 .0736225

|

gender#urbanrural |

Girl #|

Semiurban| -.0567128 .0118762 -4.78 0.000 -.0799897 -.0334359

Girl #|

Rural | -.0791137 .0118938 -6.65 0.000 -.1024252 -.0558022

|

year#gender#|

urbanrural |

2008-2009 #|

Girl #|

Semiurban| .028353 .0169211 1.68 0.094 -.0048118 .0615179

2008-2009 #|

Girl #|

Rural | .0187871 .0170804 1.10 0.271 -.01469 .0522641

2010-2011 #|

Girl #|

Semiurban| .0312466 .0170806 1.83 0.067 -.0022308 .0647239

2010-2011 #|

Girl #|

Rural | .0506094 .0173891 2.91 0.004 .0165273 .0846915

2013 #|

Girl #|

Semiurban| .0523351 .0174087 3.01 0.003 .0182146 .0864556

2013 #|

Girl #|

Rural | .0611697 .0177961 3.44 0.001 .02629 .0960493

2015 #|

Girl #|

Semiurban| .0340653 .0211569 1.61 0.107 -.0074014 .0755321

2015 #|

Girl #|

Rural | .0263287 .0220513 1.19 0.232 -.0168911 .0695485

2017 #|

Girl #|

Semiurban| .0421177 .0188934 2.23 0.026 .0050873 .0791481

2017 #|

Girl #|

Rural | -.0028847 .0194765 -0.15 0.882 -.041058 .0352886

2019 #|

Girl #|

Semiurban| .0053888 .0179041 0.30 0.763 -.0297026 .0404801

2019 #|

Girl #|

Rural | .0213155 .0189188 1.13 0.260 -.0157647 .0583958

|

school_level#gender#|

urbanrural |

Upper #|

Girl #|

Semiurban| .0456217 .0214399 2.13 0.033 .0036003 .0876431

Upper #|

Girl #|

Rural | .0725956 .0223187 3.25 0.001 .0288518 .1163393

|

year#school_level#|

gender#urbanrural |

2008-2009 #|

Upper #|

Girl #|

Semiurban| -.004954 .0308824 -0.16 0.873 -.0654825 .0555744

2008-2009 #|

Upper #|

Girl #|

Rural | -.022048 .0320471 -0.69 0.491 -.0848591 .0407631

2010-2011 #|

Upper #|

Girl #|

Semiurban| -.0405891 .0312158 -1.30 0.194 -.101771 .0205929

2010-2011 #|

Upper #|

Girl #|

Rural | -.063216 .0331961 -1.90 0.057 -.1282792 .0018472

2013 #|

Upper #|

Girl #|

Semiurban| -.0578395 .0316059 -1.83 0.067 -.119786 .004107

2013 #|

Upper #|

Girl #|

Rural | -.1029787 .0336197 -3.06 0.002 -.1688722 -.0370852

2015 #|

Upper #|

Girl #|

Semiurban| -.0029674 .0347345 -0.09 0.932 -.0710457 .0651109

2015 #|

Upper #|

Girl #|

Rural | -.0420575 .0373049 -1.13 0.260 -.1151738 .0310589

2017 #|

Upper #|

Girl #|

Semiurban| -.0444345 .0342259 -1.30 0.194 -.1115161 .022647

2017 #|

Upper #|

Girl #|

Rural | -.0341694 .0366638 -0.93 0.351 -.1060291 .0376903

2019 #|

Upper #|

Girl #|

Semiurban| -.0044534 .0321887 -0.14 0.890 -.0675421 .0586353

2019 #|

Upper #|

Girl #|

Rural | -.0154143 .0351465 -0.44 0.661 -.0843003 .0534716

|

1.education | -.0528124 .0015624 -33.80 0.000 -.0558747 -.0497502

_cons | 1.830889 .0040381 453.40 0.000 1.822975 1.838804

-----------------------------------------------------------------------------------------

. testparm school_level#gender#urbanrural

( 1) [burnout]20.school_level#2.gender#2.urbanrural = 0

( 2) [burnout]20.school_level#2.gender#3.urbanrural = 0

chi2( 2) = 12.99

Prob > chi2 = 0.0015

. margins year#school_level#gender

Predictive margins Number of obs = 897,982

Model VCE : OIM

Expression : Predicted mean burnout, predict()

------------------------------------------------------------------------------------------

| Delta-method

| Margin Std. Err. z P>|z| [95% Conf. Interval]

-------------------------+----------------------------------------------------------------

year#school_level#|

gender |

2006-2007 #|

Perusopetus 8. ja 9. lk #|

Poika | 1.813869 .0031896 568.68 0.000 1.807617 1.82012

2006-2007 #|

Perusopetus 8. ja 9. lk #|

Girl | 1.891388 .0031863 593.61 0.000 1.885143 1.897633

2006-2007#Upper#Poika | 1.804433 .0048244 374.02 0.000 1.794977 1.813888

2006-2007#Upper#Girl | 2.001379 .0041613 480.95 0.000 1.993223 2.009535

2008-2009 #|

Perusopetus 8. ja 9. lk #|

Poika | 1.818634 .0031985 568.60 0.000 1.812365 1.824903

2008-2009 #|

Perusopetus 8. ja 9. lk #|

Girl | 1.916696 .0031862 601.55 0.000 1.910451 1.922941

2008-2009#Upper#Poika | 1.786218 .0049866 358.21 0.000 1.776445 1.795992

2008-2009#Upper#Girl | 2.007051 .0042359 473.82 0.000 1.998749 2.015353

2010-2011 #|

Perusopetus 8. ja 9. lk #|

Poika | 1.797342 .0032604 551.27 0.000 1.790952 1.803733

2010-2011 #|

Perusopetus 8. ja 9. lk #|

Girl | 1.903462 .0032549 584.79 0.000 1.897082 1.909841

2010-2011#Upper#Poika | 1.738439 .0050832 342.00 0.000 1.728476 1.748402

2010-2011#Upper#Girl | 1.970951 .0043567 452.39 0.000 1.962412 1.97949

2013 #|

Perusopetus 8. ja 9. lk #|

Poika | 1.760027 .0033718 521.98 0.000 1.753418 1.766636

2013 #|

Perusopetus 8. ja 9. lk #|

Girl | 1.905892 .0033569 567.75 0.000 1.899313 1.912471

2013#Upper#Poika | 1.716953 .0051065 336.23 0.000 1.706944 1.726961

2013#Upper#Girl | 1.987353 .0043943 452.26 0.000 1.978741 1.995966

2015 #|

Perusopetus 8. ja 9. lk #|

Poika | 1.756349 .0046577 377.08 0.000 1.74722 1.765478

2015 #|

Perusopetus 8. ja 9. lk #|

Girl | 1.947776 .0045903 424.33 0.000 1.938779 1.956773

2015#Upper#Poika | 1.761293 .0057392 306.89 0.000 1.750045 1.772542

2015#Upper#Girl | 2.059995 .0047983 429.32 0.000 2.050591 2.069399

2017 #|

Perusopetus 8. ja 9. lk #|

Poika | 1.693619 .0040488 418.30 0.000 1.685684 1.701555

2017 #|

Perusopetus 8. ja 9. lk #|

Girl | 1.962933 .0038779 506.19 0.000 1.955333 1.970534

2017#Upper#Poika | 1.70606 .0061326 278.20 0.000 1.69404 1.71808

2017#Upper#Girl | 2.057897 .0051302 401.13 0.000 2.047842 2.067952

2019 #|

Perusopetus 8. ja 9. lk #|

Poika | 1.713397 .0036487 469.59 0.000 1.706246 1.720548

2019 #|

Perusopetus 8. ja 9. lk #|

Girl | 2.058945 .0035112 586.40 0.000 2.052063 2.065827

2019#Upper#Poika | 1.731048 .0054587 317.12 0.000 1.720349 1.741747

2019#Upper#Girl | 2.135601 .0045066 473.89 0.000 2.126768 2.144433

------------------------------------------------------------------------------------------

.

. marginsplot

glm burnout i.year##i.school_level##i.gender##i.unemployed i.urbanrural i.education,family(ga

> ussian) link(identity)

Iteration 0: log likelihood = -826530.96

Generalized linear models No. of obs = 771,564

Optimization : ML Residual df = 771,489

Scale parameter = .4989258

Deviance = 384915.7444 (1/df) Deviance = .4989258

Pearson = 384915.7444 (1/df) Pearson = .4989258

Variance function: V(u) = 1 [Gaussian]

Link function : g(u) = u [Identity]

AIC = 2.142676

Log likelihood = -826530.9552 BIC = -1.01e+07

------------------------------------------------------------------------------------------

| OIM

burnout | Coef. Std. Err. z P>|z| [95% Conf. Interval]

-------------------------+----------------------------------------------------------------

year |

2008-2009 | .0064672 .0050986 1.27 0.205 -.0035259 .0164604

2010-2011 | -.0190515 .0052404 -3.64 0.000 -.0293225 -.0087804

2013 | -.0545602 .0052994 -10.30 0.000 -.0649468 -.0441736

2015 | -.0653936 .0066131 -9.89 0.000 -.078355 -.0524321

2017 | -.1212319 .0059519 -20.37 0.000 -.1328974 -.1095663

|

school_level |

Upper | .000476 .0063849 0.07 0.941 -.0120382 .0129902

|

year#school_level |

2008-2009#Upper | -.0220028 .0091995 -2.39 0.017 -.0400335 -.0039722

2010-2011#Upper | -.0495583 .009424 -5.26 0.000 -.0680289 -.0310877

2013#Upper | -.0356026 .0094021 -3.79 0.000 -.0540304 -.0171748

2015#Upper | .012862 .0107497 1.20 0.231 -.0082069 .0339309

2017#Upper | .0144278 .0106469 1.36 0.175 -.0064398 .0352954

|

gender |

Girl | .0776219 .0050569 15.35 0.000 .0677105 .0875333

|

year#gender |

2008-2009#Girl | .0178408 .0072364 2.47 0.014 .0036578 .0320238

2010-2011#Girl | .0259172 .0074438 3.48 0.000 .0113277 .0405068

2013#Girl | .0567993 .0075208 7.55 0.000 .0420589 .0715397

2015#Girl | .1102622 .0093563 11.78 0.000 .0919243 .1286002

2017#Girl | .1723477 .0083686 20.59 0.000 .1559456 .1887498

|

school_level#gender |

Upper#Girl | .1204275 .0086585 13.91 0.000 .1034572 .1373979

|

year#school_level#|

gender |

2008-2009#Upper#Girl | .0036168 .0124466 0.29 0.771 -.020778 .0280116

2010-2011#Upper#Girl | .0044408 .0127694 0.35 0.728 -.0205867 .0294683

2013#Upper#Girl | .0115156 .0127579 0.90 0.367 -.0134894 .0365205

2015#Upper#Girl | -.0078956 .0145567 -0.54 0.588 -.0364262 .0206351

2017#Upper#Girl | -.0239709 .0143323 -1.67 0.094 -.0520616 .0041198

|

unemployed |

2 | .1227833 .0078403 15.66 0.000 .1074166 .13815

3 | .4258517 .0197087 21.61 0.000 .3872233 .4644802

|

year#unemployed |

2008-2009#2 | -.0277416 .0109109 -2.54 0.011 -.0491266 -.0063567

2008-2009#3 | -.0678659 .0270014 -2.51 0.012 -.1207877 -.014944

2010-2011#2 | -.0380629 .0107337 -3.55 0.000 -.0591006 -.0170252

2010-2011#3 | -.0398695 .0262817 -1.52 0.129 -.0913807 .0116417

2013#2 | -.0314582 .0110324 -2.85 0.004 -.0530814 -.0098351

2013#3 | -.1181402 .0272994 -4.33 0.000 -.1716461 -.0646344

2015#2 | -.0470733 .0130275 -3.61 0.000 -.0726068 -.0215398

2015#3 | .0385236 .0308069 1.25 0.211 -.0218568 .098904

2017#2 | -.0499316 .0120975 -4.13 0.000 -.0736423 -.0262209

2017#3 | -.0666654 .0287853 -2.32 0.021 -.1230836 -.0102472

|

school_level#unemployed |

Upper#2 | -.0322247 .0147436 -2.19 0.029 -.0611217 -.0033278

Upper#3 | -.1358342 .0403777 -3.36 0.001 -.2149731 -.0566954

|

year#school_level#|

unemployed |

2008-2009#Upper#2 | .0084678 .020729 0.41 0.683 -.0321604 .049096

2008-2009#Upper#3 | .0038216 .0565518 0.07 0.946 -.1070179 .114661

2010-2011#Upper#2 | .0175289 .0202609 0.87 0.387 -.0221817 .0572396

2010-2011#Upper#3 | .0082283 .053362 0.15 0.877 -.0963593 .1128159

2013#Upper#2 | .0111744 .020618 0.54 0.588 -.029236 .0515849

2013#Upper#3 | .0363744 .0562251 0.65 0.518 -.0738247 .1465735

2015#Upper#2 | .0379112 .0223043 1.70 0.089 -.0058045 .0816269

2015#Upper#3 | -.0138706 .0575945 -0.24 0.810 -.1267537 .0990126

2017#Upper#2 | .0439648 .0223542 1.97 0.049 .0001514 .0877782

2017#Upper#3 | .0673401 .0589915 1.14 0.254 -.0482812 .1829613

|

gender#unemployed |

Girl#2 | .0002577 .0109025 0.02 0.981 -.0211108 .0216262

Girl#3 | -.1653512 .0281257 -5.88 0.000 -.2204767 -.1102258

|

year#gender#unemployed |

2008-2009#Girl#2 | .0067944 .0151833 0.45 0.655 -.0229642 .0365531

2008-2009#Girl#3 | .1368152 .0385855 3.55 0.000 .061189 .2124415

2010-2011#Girl#2 | .0165041 .0149786 1.10 0.271 -.0128535 .0458616

2010-2011#Girl#3 | .0596462 .0371551 1.61 0.108 -.0131764 .1324688

2013#Girl#2 | .0378561 .0153674 2.46 0.014 .0077365 .0679758

2013#Girl#3 | .1749832 .0387889 4.51 0.000 .0989585 .251008

2015#Girl#2 | .0326054 .0181019 1.80 0.072 -.0028737 .0680845

2015#Girl#3 | .0083441 .0436726 0.19 0.848 -.0772527 .0939409

2017#Girl#2 | .0639985 .0166146 3.85 0.000 .0314345 .0965626

2017#Girl#3 | .109374 .0406429 2.69 0.007 .0297154 .1890327

|

school_level#gender#|

unemployed |

Upper#Girl#2 | .0083318 .0195486 0.43 0.670 -.0299827 .0466463

Upper#Girl#3 | .0274721 .0546531 0.50 0.615 -.079646 .1345901

|

year#school_level#|

gender#unemployed |

2008-2009#Upper#Girl#2 | -.0044339 .0274505 -0.16 0.872 -.0582359 .049368

2008-2009#Upper#Girl#3 | -.0266281 .0757825 -0.35 0.725 -.1751591 .1219029

2010-2011#Upper#Girl#2 | .0037639 .0269595 0.14 0.889 -.0490758 .0566036

2010-2011#Upper#Girl#3 | .0406448 .0719942 0.56 0.572 -.1004611 .1817508

2013#Upper#Girl#2 | -.0146029 .0274307 -0.53 0.594 -.068366 .0391603

2013#Upper#Girl#3 | -.0487295 .0752924 -0.65 0.517 -.1962999 .0988408

2015#Upper#Girl#2 | -.031976 .0296462 -1.08 0.281 -.0900814 .0261295

2015#Upper#Girl#3 | -.0115766 .0772215 -0.15 0.881 -.1629279 .1397747

2017#Upper#Girl#2 | -.0396352 .0294618 -1.35 0.179 -.0973792 .0181087

2017#Upper#Girl#3 | -.0684665 .0784723 -0.87 0.383 -.2222694 .0853365

|

urbanrural |

Semiurban| -.0140404 .0022031 -6.37 0.000 -.0183584 -.0097224

Rural | -.0180753 .0023121 -7.82 0.000 -.0226069 -.0135438

|

1.education | -.0370187 .0016716 -22.15 0.000 -.040295 -.0337424

_cons | 1.8036 .0036905 488.72 0.000 1.796366 1.810833

------------------------------------------------------------------------------------------

. testparm year#school_level#gender#unemployed

( 1) [burnout]7.year#20.school_level#2.gender#2.unemployed = 0

( 2) [burnout]7.year#20.school_level#2.gender#3.unemployed = 0

( 3) [burnout]8.year#20.school_level#2.gender#2.unemployed = 0

( 4) [burnout]8.year#20.school_level#2.gender#3.unemployed = 0

( 5) [burnout]9.year#20.school_level#2.gender#2.unemployed = 0

( 6) [burnout]9.year#20.school_level#2.gender#3.unemployed = 0

( 7) [burnout]10.year#20.school_level#2.gender#2.unemployed = 0

( 8) [burnout]10.year#20.school_level#2.gender#3.unemployed = 0

( 9) [burnout]11.year#20.school_level#2.gender#2.unemployed = 0

(10) [burnout]11.year#20.school_level#2.gender#3.unemployed = 0

chi2( 10) = 6.10

Prob > chi2 = 0.8067

.

. testparm school_level#gender#unemployed

( 1) [burnout]20.school_level#2.gender#2.unemployed = 0

( 2) [burnout]20.school_level#2.gender#3.unemployed = 0

chi2( 2) = 0.41

Prob > chi2 = 0.8162

.

. testparm year#gender#unemployed

( 1) [burnout]7.year#2.gender#2.unemployed = 0

( 2) [burnout]7.year#2.gender#3.unemployed = 0

( 3) [burnout]8.year#2.gender#2.unemployed = 0

( 4) [burnout]8.year#2.gender#3.unemployed = 0

( 5) [burnout]9.year#2.gender#2.unemployed = 0

( 6) [burnout]9.year#2.gender#3.unemployed = 0

( 7) [burnout]10.year#2.gender#2.unemployed = 0

( 8) [burnout]10.year#2.gender#3.unemployed = 0

( 9) [burnout]11.year#2.gender#2.unemployed = 0

(10) [burnout]11.year#2.gender#3.unemployed = 0

chi2( 10) = 50.24

Prob > chi2 = 0.0000

. testparm gender#unemployed

( 1) [burnout]2.gender#2.unemployed = 0

( 2) [burnout]2.gender#3.unemployed = 0

chi2( 2) = 34.83

Prob > chi2 = 0.0000

. testparm year#school_level#unemployed

( 1) [burnout]7.year#20.school_level#2.unemployed = 0

( 2) [burnout]7.year#20.school_level#3.unemployed = 0

( 3) [burnout]8.year#20.school_level#2.unemployed = 0

( 4) [burnout]8.year#20.school_level#3.unemployed = 0

( 5) [burnout]9.year#20.school_level#2.unemployed = 0

( 6) [burnout]9.year#20.school_level#3.unemployed = 0

( 7) [burnout]10.year#20.school_level#2.unemployed = 0

( 8) [burnout]10.year#20.school_level#3.unemployed = 0

( 9) [burnout]11.year#20.school_level#2.unemployed = 0

(10) [burnout]11.year#20.school_level#3.unemployed = 0

chi2( 10) = 8.31

Prob > chi2 = 0.5988

.

. testparm school_level#unemployed

( 1) [burnout]20.school_level#2.unemployed = 0

( 2) [burnout]20.school_level#3.unemployed = 0

chi2( 2) = 15.16

Prob > chi2 = 0.0005

. testparm year#unemployed

( 1) [burnout]7.year#2.unemployed = 0

( 2) [burnout]7.year#3.unemployed = 0

( 3) [burnout]8.year#2.unemployed = 0

( 4) [burnout]8.year#3.unemployed = 0

( 5) [burnout]9.year#2.unemployed = 0

( 6) [burnout]9.year#3.unemployed = 0

( 7) [burnout]10.year#2.unemployed = 0

( 8) [burnout]10.year#3.unemployed = 0

( 9) [burnout]11.year#2.unemployed = 0

(10) [burnout]11.year#3.unemployed = 0

chi2( 10) = 58.78

Prob > chi2 = 0.0000

. testparm year#school_level#gender

( 1) [burnout]7.year#20.school_level#2.gender = 0

( 2) [burnout]8.year#20.school_level#2.gender = 0

( 3) [burnout]9.year#20.school_level#2.gender = 0

( 4) [burnout]10.year#20.school_level#2.gender = 0

( 5) [burnout]11.year#20.school_level#2.gender = 0

chi2( 5) = 6.75

Prob > chi2 = 0.2395

.

. testparm school_level#gender

( 1) [burnout]20.school_level#2.gender = 0

chi2( 1) = 193.45

Prob > chi2 = 0.0000

. testparm year#gender

( 1) [burnout]7.year#2.gender = 0

( 2) [burnout]8.year#2.gender = 0

( 3) [burnout]9.year#2.gender = 0

( 4) [burnout]10.year#2.gender = 0

( 5) [burnout]11.year#2.gender = 0

chi2( 5) = 552.54

Prob > chi2 = 0.0000

. testparm year#school_level

( 1) [burnout]7.year#20.school_level = 0

( 2) [burnout]8.year#20.school_level = 0

( 3) [burnout]9.year#20.school_level = 0

( 4) [burnout]10.year#20.school_level = 0

( 5) [burnout]11.year#20.school_level = 0

chi2( 5) = 62.43

Prob > chi2 = 0.0000

. margins school_level#unemployed

Predictive margins Number of obs = 771,564

Model VCE : OIM

Expression : Predicted mean burnout, predict()

------------------------------------------------------------------------------------------

| Delta-method

| Margin Std. Err. z P>|z| [95% Conf. Interval]

-------------------------+----------------------------------------------------------------

school_level#unemployed |

Perusopetus 8. ja 9. lk #|

1 | 1.816488 .0011834 1534.93 0.000 1.814168 1.818807

Perusopetus 8. ja 9. lk #|

2 | 1.921936 .0020095 956.43 0.000 1.917998 1.925875

Perusopetus 8. ja 9. lk #|

3 | 2.153086 .0055863 385.42 0.000 2.142137 2.164035

Upper#1 | 1.863992 .0015979 1166.53 0.000 1.86086 1.867124

Upper#2 | 1.952259 .0029549 660.70 0.000 1.946468 1.958051

Upper#3 | 2.086391 .0090818 229.73 0.000 2.068591 2.104191

------------------------------------------------------------------------------------------

. marginsplot

. glm burnout i.year i.school_level i.gender i.unemployed i.urbanrural i.education,family(gau

> ssian) link(identity)

Iteration 0: log likelihood = -828059.39

Generalized linear models No. of obs = 771,564

Optimization : ML Residual df = 771,551

Scale parameter = .5008661

Deviance = 386443.7713 (1/df) Deviance = .5008661

Pearson = 386443.7713 (1/df) Pearson = .5008661

Variance function: V(u) = 1 [Gaussian]

Link function : g(u) = u [Identity]

AIC = 2.146478

Log likelihood = -828059.3892 BIC = -1.01e+07

----------------------------------------------------------------------------------------

| OIM

burnout | Coef. Std. Err. z P>|z| [95% Conf. Interval]

-----------------------+----------------------------------------------------------------

year |

2008-2009 | .0045913 .0025669 1.79 0.074 -.0004398 .0096223

2010-2011 | -.0262807 .0025969 -10.12 0.000 -.0313706 -.0211908

2013 | -.0371627 .0026343 -14.11 0.000 -.0423258 -.0319997

2015 | -.0047251 .0030293 -1.56 0.119 -.0106625 .0012123

2017 | -.0295719 .002902 -10.19 0.000 -.0352598 -.023884

|

school_level |

Upper | .0438366 .001719 25.50 0.000 .0404674 .0472057

|

gender |

Girl | .174536 .0016204 107.71 0.000 .17136 .1777119

|

unemployed |

2 | .0998761 .0019064 52.39 0.000 .0961396 .1036126

3 | .3053423 .0047856 63.80 0.000 .2959626 .3147219

|

urbanrural |

Semiurban| -.0141969 .002207 -6.43 0.000 -.0185227 -.0098712

Rural | -.0181304 .0023162 -7.83 0.000 -.02267 -.0135908

|

1.education | -.037418 .0016739 -22.35 0.000 -.0406988 -.0341372

_cons | 1.764558 .0023151 762.19 0.000 1.760021 1.769096

----------------------------------------------------------------------------------------

. glm burnout i.year i.school_level i.gender i.unemployed i.urbanrural i.education i.Syntyper

> a,family(gaussian) link(identity)

Iteration 0: log likelihood = -349263.12

Generalized linear models No. of obs = 320,351

Optimization : ML Residual df = 320,338

Scale parameter = .5182313

Deviance = 166009.1644 (1/df) Deviance = .5182313

Pearson = 166009.1644 (1/df) Pearson = .5182313

Variance function: V(u) = 1 [Gaussian]

Link function : g(u) = u [Identity]

AIC = 2.180584

Log likelihood = -349263.1248 BIC = -3894971

------------------------------------------------------------------------------------------

| OIM

burnout | Coef. Std. Err. z P>|z| [95% Conf. Interval]

-------------------------+----------------------------------------------------------------

year |

2015 | .030104 .0031509 9.55 0.000 .0239284 .0362796

2017 | .0058101 .0030179 1.93 0.054 -.0001048 .0117249

|

school_level |

Upper | .0574919 .002689 21.38 0.000 .0522215 .0627623

|

gender |

Girl | .237778 .0025609 92.85 0.000 .2327587 .2427973

|

unemployed |

2 | .0933066 .0029439 31.69 0.000 .0875366 .0990766

3 | .262515 .0073543 35.70 0.000 .2481008 .2769292

|

urbanrural |

Semiurban| -.0129946 .0034991 -3.71 0.000 -.0198528 -.0061365

Rural | -.0178466 .0037406 -4.77 0.000 -.025178 -.0105151

|

1.education | -.0467878 .0026255 -17.82 0.000 -.0519337 -.0416419

|

Immigration |

Toinen vanhemmista ul.. | .1113951 .0052305 21.30 0.000 .1011434 .1216467

Ulkomaalaistaustainen.. | .0839381 .0098892 8.49 0.000 .0645556 .1033205

Ulkomaalaistaustainen.. | .220267 .0080083 27.50 0.000 .204571 .2359629

|

_cons | 1.68112 .0031708 530.19 0.000 1.674906 1.687335

------------------------------------------------------------------------------------------

. glm burnout i.year##i.school_level##i.gender##i.Immigration Immigration i.unemployed i.kunta

> ryhma i.education,family(gaussian) link(identity)

note: Immigration omitted because of collinearity

Iteration 0: log likelihood = -348640.23

Generalized linear models No. of obs = 320,351

Optimization : ML Residual df = 320,298

Scale parameter = .5162843

Deviance = 165364.8387 (1/df) Deviance = .5162843

Pearson = 165364.8387 (1/df) Pearson = .5162843

Variance function: V(u) = 1 [Gaussian]

Link function : g(u) = u [Identity]

AIC = 2.176945

Log likelihood = -348640.2314 BIC = -3895108

------------------------------------------------------------------------------------------

| OIM

burnout | Coef. Std. Err. z P>|z| [95% Conf. Interval]

-------------------------+----------------------------------------------------------------

year |

2015 | -.0157306 .006207 -2.53 0.011 -.0278962 -.003565

2017 | -.0718612 .0056861 -12.64 0.000 -.0830057 -.0607167

|

school_level |

Upper | -.0291696 .0064319 -4.54 0.000 -.041776 -.0165632

|

year#school_level |

2015#Upper | .0562693 .0101565 5.54 0.000 .0363629 .0761757

2017#Upper | .0559385 .0101199 5.53 0.000 .0361039 .0757731

|

gender |

Girl | .1577473 .0050771 31.07 0.000 .1477964 .1676983

|

year#gender |

2015#Girl | .054368 .0086861 6.26 0.000 .0373436 .0713925

2017#Girl | .1212913 .007898 15.36 0.000 .1058116 .1367711

|

school_level#gender |

Upper#Girl | .1189968 .0086443 13.77 0.000 .1020543 .1359393

|

year#school_level#|

gender |

2015#Upper#Girl | -.0271834 .013646 -1.99 0.046 -.0539291 -.0004378

2017#Upper#Girl | -.0370592 .0135105 -2.74 0.006 -.0635394 -.0105791

|

Immigration |

Toinen vanhemmista ul.. | .1120383 .0147471 7.60 0.000 .0831346 .1409421

Ulkomaalaistaustainen.. | .1224236 .0266545 4.59 0.000 .0701817 .1746655

Ulkomaalaistaustainen.. | .3228385 .0198113 16.30 0.000 .284009 .3616679

|

year#Immigration |

2015 #|

Toinen vanhemmista ul.. | -.0188419 .024334 -0.77 0.439 -.0665355 .0288518

2015 #|

Ulkomaalaistaustainen.. | -.0478469 .0436278 -1.10 0.273 -.1333557 .037662

2015 #|

Ulkomaalaistaustainen.. | .1432405 .02959 4.84 0.000 .0852452 .2012357

2017 #|

Toinen vanhemmista ul.. | -.0273318 .0217318 -1.26 0.209 -.0699254 .0152618

2017 #|

Ulkomaalaistaustainen.. | -.0023432 .0408178 -0.06 0.954 -.0823446 .0776582

2017 #|

Ulkomaalaistaustainen.. | .05431 .0284644 1.91 0.056 -.0014792 .1100992

|

school_level#Immigration |

Upper #|

Toinen vanhemmista ul.. | -.003649 .0272195 -0.13 0.893 -.0569983 .0497004

Upper #|

Ulkomaalaistaustainen.. | -.0054078 .049762 -0.11 0.913 -.1029396 .092124

Upper #|

Ulkomaalaistaustainen.. | -.0743822 .0434249 -1.71 0.087 -.1594935 .0107291

|

year#school_level#|

Immigration |

2015 #|

Upper #|

Toinen vanhemmista ul.. | .0218872 .0411036 0.53 0.594 -.0586744 .1024489

2015 #|

Upper #|

Ulkomaalaistaustainen.. | .0655708 .0757038 0.87 0.386 -.0828059 .2139474

2015 #|

Upper #|

Ulkomaalaistaustainen.. | -.1790687 .0615714 -2.91 0.004 -.2997464 -.0583909

2017 #|

Upper #|

Toinen vanhemmista ul.. | .0499328 .0402645 1.24 0.215 -.0289841 .1288497

2017 #|

Upper #|

Ulkomaalaistaustainen.. | .0552288 .0755612 0.73 0.465 -.0928686 .2033261

2017 #|

Upper #|

Ulkomaalaistaustainen.. | -.0479827 .0642925 -0.75 0.455 -.1739936 .0780282

|

gender#Immigration |

Girl #|

Toinen vanhemmista ul.. | .0110774 .0201448 0.55 0.582 -.0284058 .0505605

Girl #|

Ulkomaalaistaustainen.. | -.1174706 .0384077 -3.06 0.002 -.1927483 -.042193

Girl #|

Ulkomaalaistaustainen.. | -.22749 .0304854 -7.46 0.000 -.2872403 -.1677398

|

year#gender#|

Immigration |

2015 #|

Girl #|

Toinen vanhemmista ul.. | -.0036665 .0332727 -0.11 0.912 -.0688799 .0615468

2015 #|

Girl #|

Ulkomaalaistaustainen.. | .0234118 .0610198 0.38 0.701 -.0961848 .1430084

2015 #|

Girl #|

Ulkomaalaistaustainen.. | -.1417571 .0475157 -2.98 0.003 -.2348861 -.0486281

2017 #|

Girl #|

Toinen vanhemmista ul.. | .0472508 .0296642 1.59 0.111 -.0108899 .1053915

2017 #|

Girl #|

Ulkomaalaistaustainen.. | .086226 .0567907 1.52 0.129 -.0250818 .1975338

2017 #|

Girl #|

Ulkomaalaistaustainen.. | -.1195847 .0438591 -2.73 0.006 -.2055469 -.0336225

|

school_level#gender#|

Immigration |

Upper #|

Girl #|

Toinen vanhemmista ul.. | -.0158436 .0359119 -0.44 0.659 -.0862296 .0545423

Upper #|

Girl #|

Ulkomaalaistaustainen.. | .0884261 .0679784 1.30 0.193 -.0448091 .2216612

Upper #|

Girl #|

Ulkomaalaistaustainen.. | .0442379 .0605696 0.73 0.465 -.0744764 .1629522

|

year#school_level#|

gender#Immigration |

2015 #|

Upper #|

Girl #|

Toinen vanhemmista ul.. | -.004757 .0544663 -0.09 0.930 -.111509 .1019951

2015 #|

Upper #|

Girl #|

Ulkomaalaistaustainen.. | .0398708 .1020502 0.39 0.696 -.160144 .2398855

2015 #|

Upper #|

Girl #|

Ulkomaalaistaustainen.. | .1023527 .0879228 1.16 0.244 -.0699728 .2746781

2017 #|

Upper #|

Girl #|

Toinen vanhemmista ul.. | -.0469263 .0532809 -0.88 0.378 -.1513549 .0575024

2017 #|

Upper #|

Girl #|

Ulkomaalaistaustainen.. | -.1923509 .1009089 -1.91 0.057 -.3901287 .005427

2017 #|

Upper #|

Girl #|

Ulkomaalaistaustainen.. | .0075554 .0890797 0.08 0.932 -.1670377 .1821485

|

Immigration | 0 (omitted)

|

unemployed |

2 | .093952 .0029391 31.97 0.000 .0881915 .0997126

3 | .2580143 .0073449 35.13 0.000 .2436186 .2724099

|

urbanrural |

Semiurban| -.0135538 .0034929 -3.88 0.000 -.0203998 -.0067078

Rural | -.0184979 .003734 -4.95 0.000 -.0258164 -.0111793

|

1.education | -.045665 .0026216 -17.42 0.000 -.0508032 -.0405268

_cons | 1.729621 .0040687 425.10 0.000 1.721646 1.737595

------------------------------------------------------------------------------------------

. testparm year#school_level#gender#Immigration

( 1) [burnout]10.year#20.school_level#2.gender#2.Immigration = 0

( 2) [burnout]10.year#20.school_level#2.gender#3.Immigration = 0

( 3) [burnout]10.year#20.school_level#2.gender#4.Immigration = 0

( 4) [burnout]11.year#20.school_level#2.gender#2.Immigration = 0

( 5) [burnout]11.year#20.school_level#2.gender#3.Immigration = 0

( 6) [burnout]11.year#20.school_level#2.gender#4.Immigration = 0

chi2( 6) = 7.85

Prob > chi2 = 0.2490

.

. testparm school_level#gender#Immigration

( 1) [burnout]20.school_level#2.gender#2.Immigration = 0

( 2) [burnout]20.school_level#2.gender#3.Immigration = 0

( 3) [burnout]20.school_level#2.gender#4.Immigration = 0

chi2( 3) = 2.45

Prob > chi2 = 0.4849

.

. testparm year#gender#Immigration

( 1) [burnout]10.year#2.gender#2.Immigration = 0

( 2) [burnout]10.year#2.gender#3.Immigration = 0

( 3) [burnout]10.year#2.gender#4.Immigration = 0

( 4) [burnout]11.year#2.gender#2.Immigration = 0

( 5) [burnout]11.year#2.gender#3.Immigration = 0

( 6) [burnout]11.year#2.gender#4.Immigration = 0

chi2( 6) = 17.21

Prob > chi2 = 0.0086

.

. testparm gender#Immigration

( 1) [burnout]2.gender#2.Immigration = 0

( 2) [burnout]2.gender#3.Immigration = 0

( 3) [burnout]2.gender#4.Immigration = 0

chi2( 3) = 64.98

Prob > chi2 = 0.0000

. testparm year#school_level#Immigration

( 1) [burnout]10.year#20.school_level#2.Immigration = 0

( 2) [burnout]10.year#20.school_level#3.Immigration = 0

( 3) [burnout]10.year#20.school_level#4.Immigration = 0

( 4) [burnout]11.year#20.school_level#2.Immigration = 0

( 5) [burnout]11.year#20.school_level#3.Immigration = 0

( 6) [burnout]11.year#20.school_level#4.Immigration = 0

chi2( 6) = 11.54

Prob > chi2 = 0.0730

.

. testparm school_level#Immigration

( 1) [burnout]20.school_level#2.Immigration = 0

( 2) [burnout]20.school_level#3.Immigration = 0

( 3) [burnout]20.school_level#4.Immigration = 0

chi2( 3) = 2.95

Prob > chi2 = 0.4002

. testparm year#Immigration

( 1) [burnout]10.year#2.Immigration = 0

( 2) [burnout]10.year#3.Immigration = 0

( 3) [burnout]10.year#4.Immigration = 0

( 4) [burnout]11.year#2.Immigration = 0

( 5) [burnout]11.year#3.Immigration = 0

( 6) [burnout]11.year#4.Immigration = 0

chi2( 6) = 27.34

Prob > chi2 = 0.0001

. testparm year#school_level#gender

( 1) [burnout]10.year#20.school_level#2.gender = 0

( 2) [burnout]11.year#20.school_level#2.gender = 0

chi2( 2) = 8.45

Prob > chi2 = 0.0146

.

. testparm school_level#gender

( 1) [burnout]20.school_level#2.gender = 0

chi2( 1) = 189.50

Prob > chi2 = 0.0000

. testparm year#gender

( 1) [burnout]10.year#2.gender = 0

( 2) [burnout]11.year#2.gender = 0

chi2( 2) = 236.12

Prob > chi2 = 0.0000

. testparm year#school_level

( 1) [burnout]10.year#20.school_level = 0

( 2) [burnout]11.year#20.school_level = 0

chi2( 2) = 43.75

Prob > chi2 = 0.0000
