# Supplementary material for: School burnout trends and sociodemographic factors in Finland 2006–2019
Source: Soc Psychiatry Psychiatr Epidemiol. 2022 Mar 22;57(8):1659–69. doi: 10.1007/s00127-022-02268-0 (PMC9288953; doi:10.1007/s00127-022-02268-0)
Supplement: Supplementary file 3 — Supplementary file3 (DOCX 1227 KB) [file 127_2022_2268_MOESM3_ESM.docx]

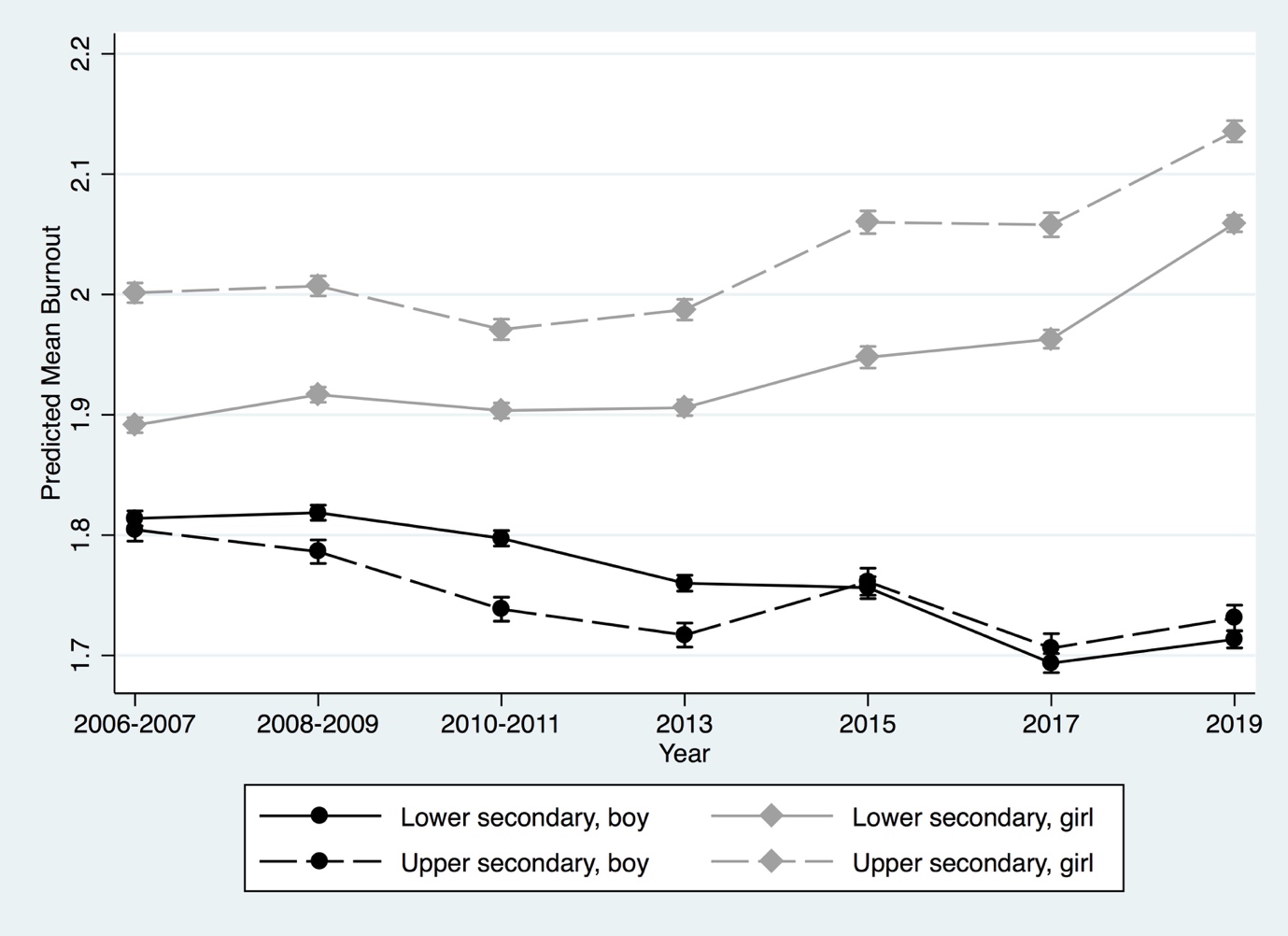


Online Resource 3. School burnout by gender and school level in Finland between 2006 and 2019 (the interaction in the adjusted model in Table 3).


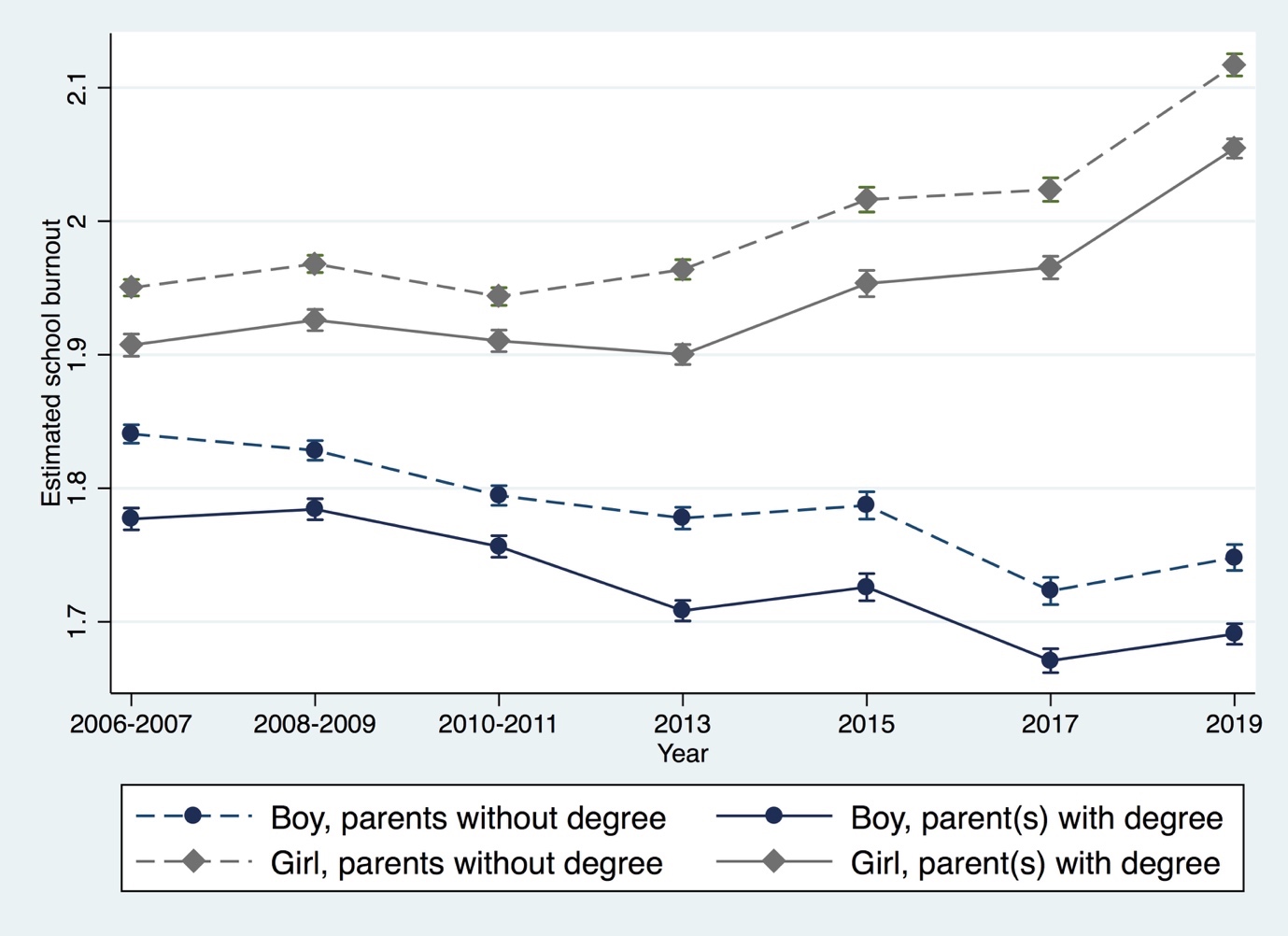


Online Resource 4. School burnout by gender and parental education in secondary school in Finland between 2006 and 2019 (the interaction in the adjusted model 2 in Table 3).


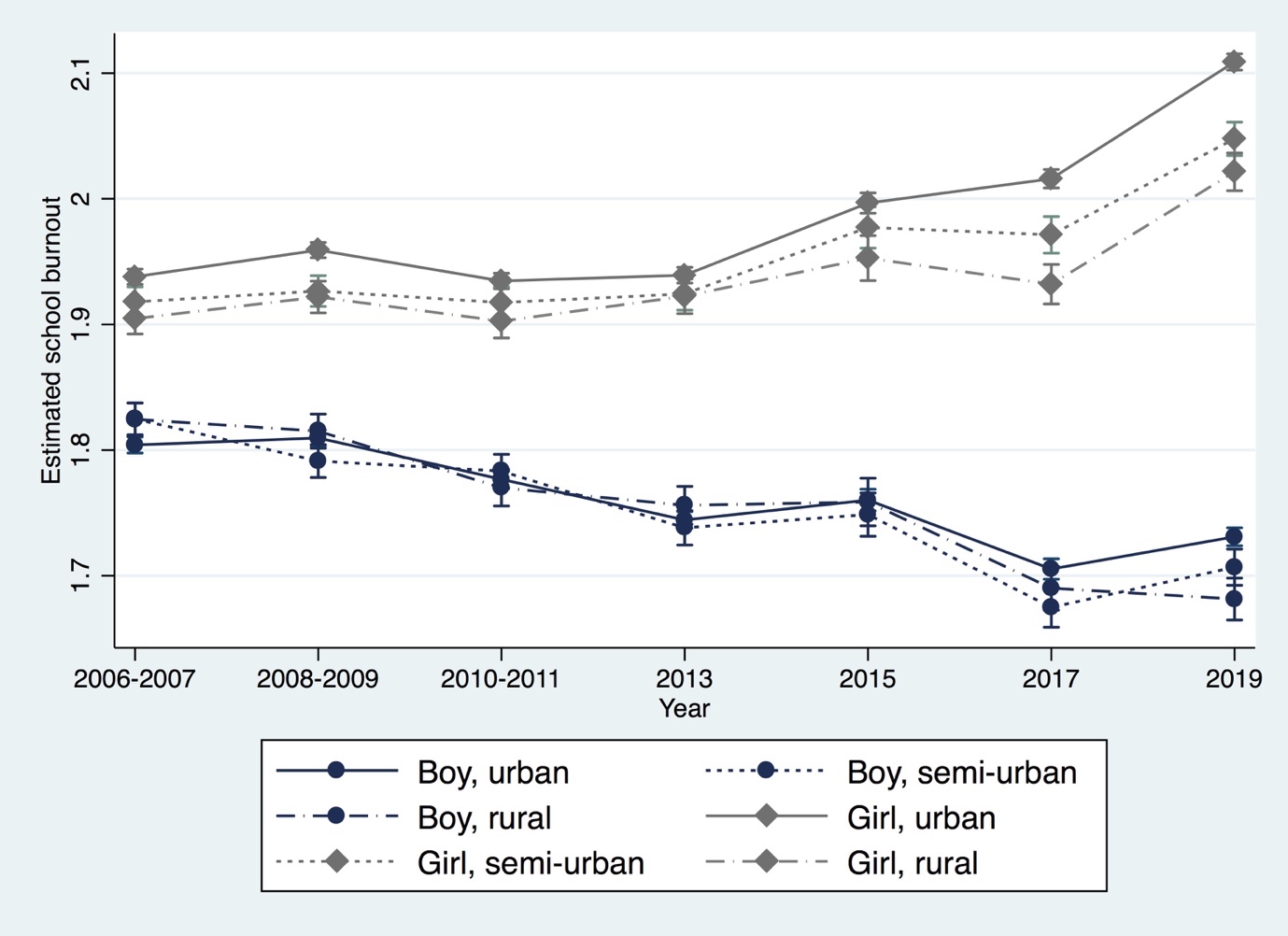


Online Resource 5. School burnout by gender and urban-rural classification in secondary school in Finland between 2006 and 2019 (the interaction in the adjusted model 2 in Table 3).


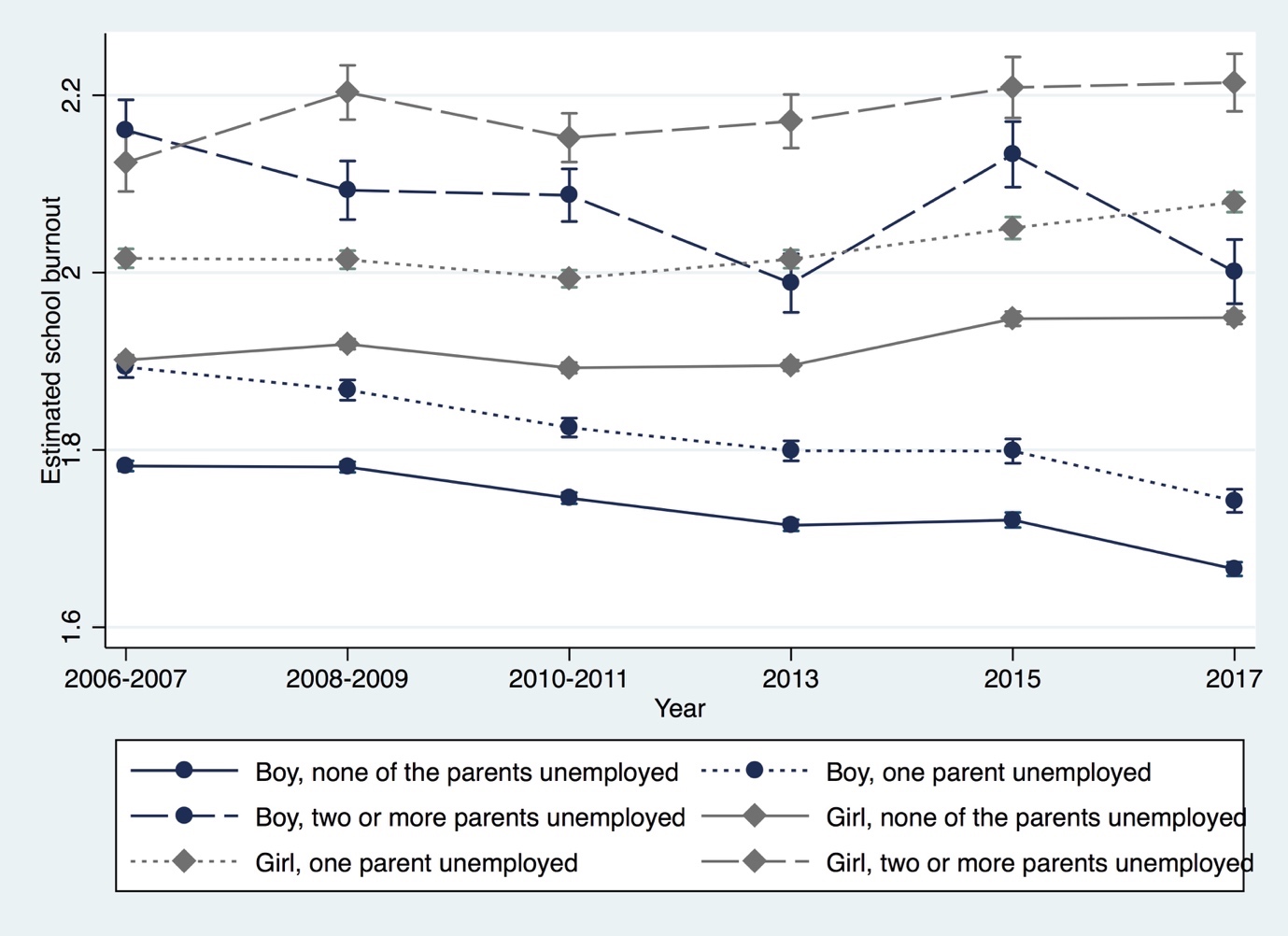


Online Resource 6. School burnout gender and parental employment status in secondary school in Finland between 2006 and 2019 (the interaction in the adjusted model in Table 4).


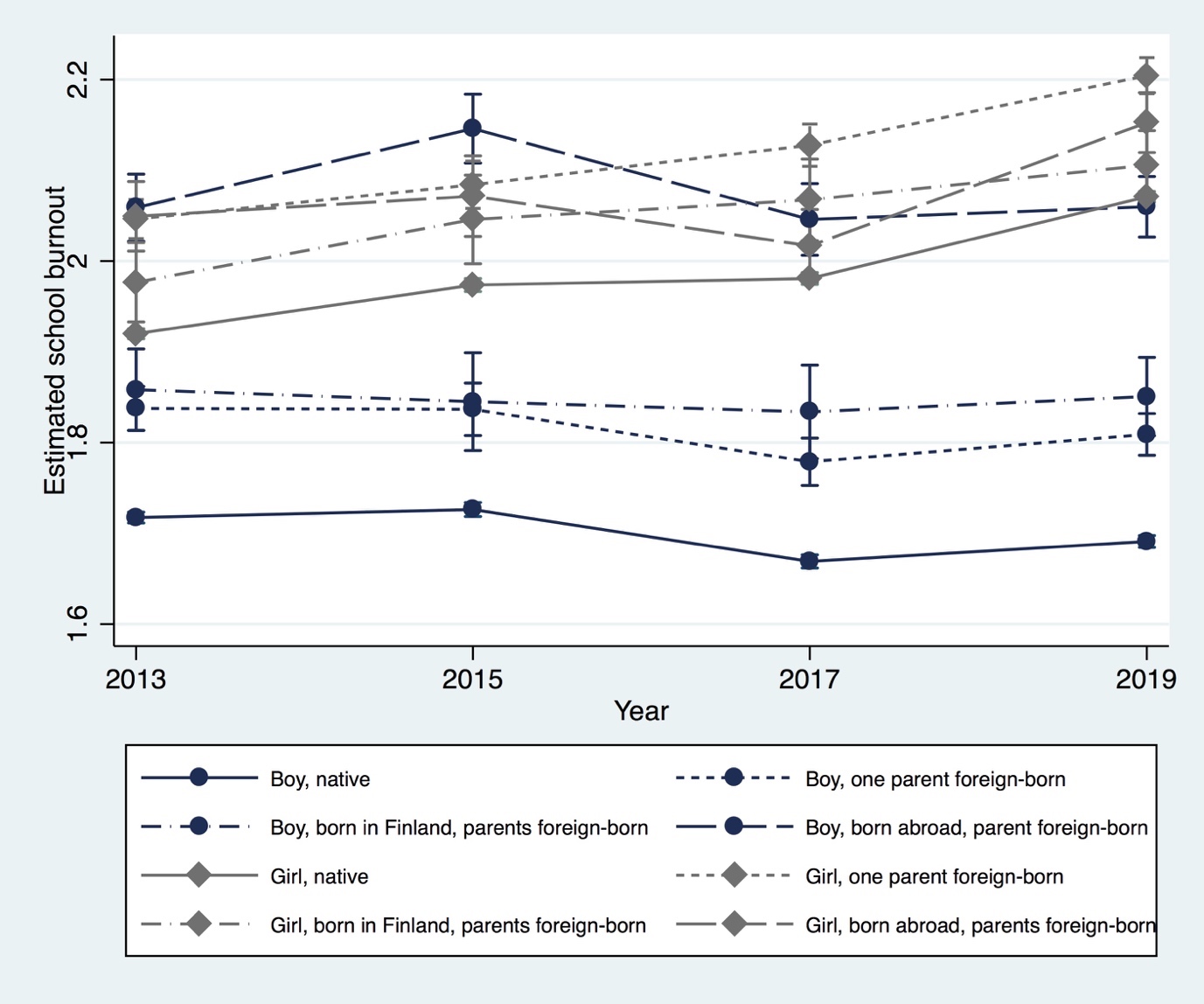


Online Resource 7. School burnout by gender and immigration status in secondary school in Finland between 2013 and 2019 (the interaction in the adjusted model in Table 5).


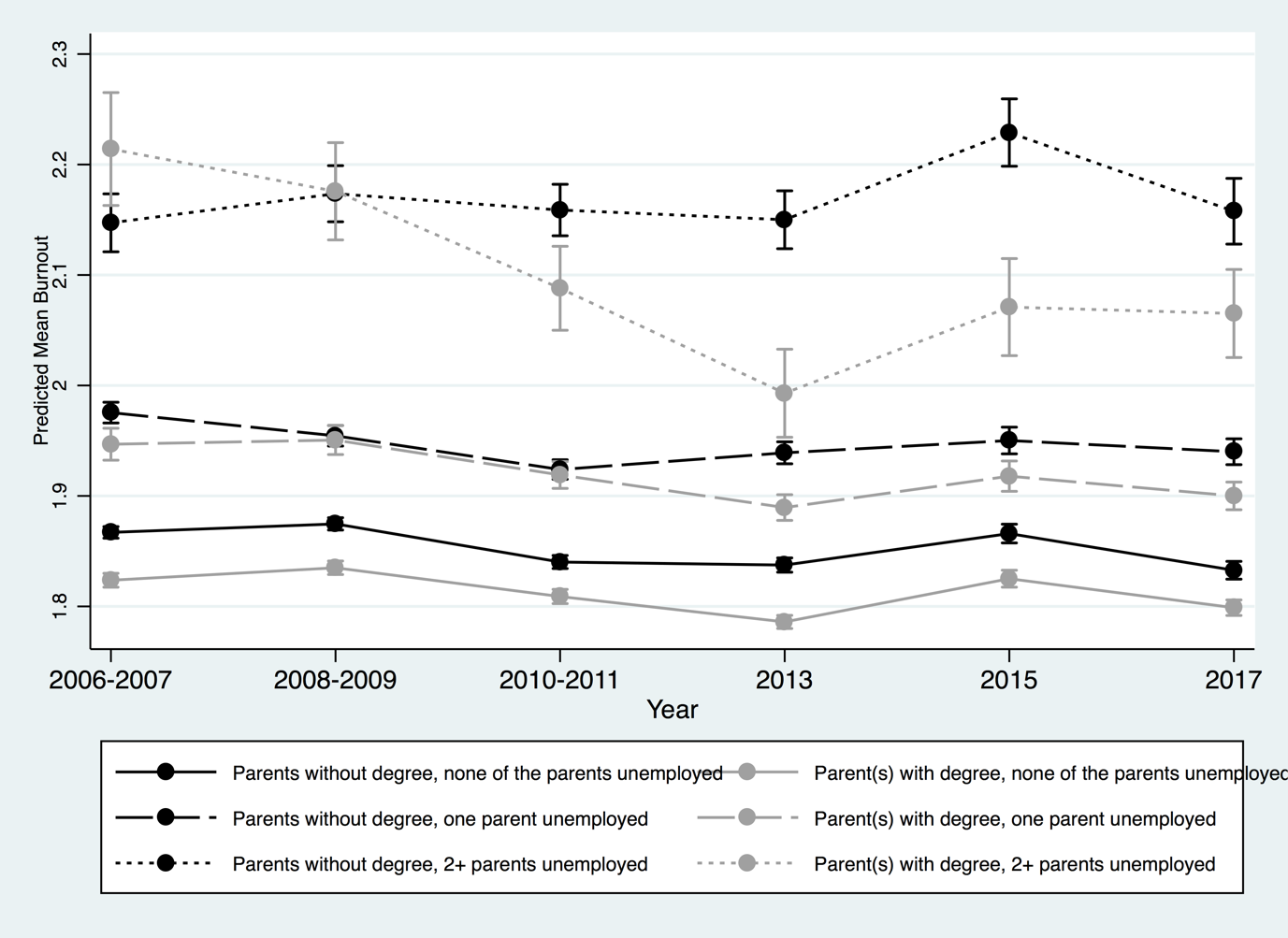


Online Resource 8. School burnout by parental education and employment in secondary school in Finland between 2006 and 2017.

Online Resource 9. School burnout by parental education and immigration status in secondary school Finland.
